# Synthesis, Characterization and Photodynamic Activity against Bladder Cancer Cells of Novel Triazole-Porphyrin Derivatives

**DOI:** 10.3390/molecules25071607

**Published:** 2020-03-31

**Authors:** Ana T. P. C. Gomes, Rosa Fernandes, Carlos F. Ribeiro, João P. C. Tomé, Maria G. P. M. S. Neves, Fernando de C. da Silva, Vítor F. Ferreira, José A. S. Cavaleiro

**Affiliations:** 1 LAQV-REQUIMTE, Department of Chemistry, University of Aveiro, 3810-193 Aveiro, Portugal; gneves@ua.pt; 2 Coimbra Institute for Clinical and Biomedical Research (iCBR), Faculty of Medicine, University of Coimbra, 3000-548 Coimbra, Portugal; cribeiro@fmed.uc.pt; 3 Center for Innovative Biomedicine and Biotechnology (CIBB), University of Coimbra, 3004-504 Coimbra, Portugal; 4 CQE, Departamento de Engenharia Química, Instituto Superior Técnico, Universidade de Lisboa, 1049-001 Lisboa, Portugal; jtome@tecnico.ulisboa.pt; 5 Departamento de Química Orgânica, Instituto de Química, Universidade Federal Fluminense, Niterói, RJ 24020-150, Brazil; gqofernando@yahoo.com.br; 6 Departamento de Tecnologia Farmacêutica, Faculdade de Farmácia, Universidade Federal Fluminense, Niterói, RJ 24241-000, Brazil; vitorferreira@id.uff.br

**Keywords:** porphyrin, triazole, polyvinylpyrrolidone, photodynamic therapy, human bladder cancer cells, human retinal pigment epithelial cells

## Abstract

Novel triazole-porphyrin derivatives (TZ-PORs) were synthesized through the Heck reaction and then incorporated into polyvinylpyrrolidone (PVP) micelles. After verifying that this incorporation did not compromise the photophysical and chemical features of TZ-PORs as photosensitizers, the phototoxicity of the formulations towards cancer cells was screened. Biological studies show high photodynamic activity of all PVP-TZ-POR formulations against a bladder cancer cell line with a particular highlight to PVP-TZ-POR **7e** and **7f** that are able to significantly reduce HT-1376 cell viability, while they had no effect on control ARPE-19 cells.

## 1. Introduction

The unique physical-chemical properties displayed by porphyrins are responsible for the promising potentialities of these compounds as catalysts, sensors, in the development of electronic devices and for solar cells production [1,2,3]. These compounds are considered to be highly relevant in the medicinal field due to their high ability to act as photosensitizers in photodynamic therapy (PDT) [4]. In fact, porphyrins and analogues are special candidates for the management of oncological diseases by following the PDT therapy [5,6,7,8,9,10,11,12,13]. PDT is centered in a photooxidation process occurring in target tissues through three key components: photosensitizer (PS), oxygen, and light (sources emitting within the absorption spectrum of the PS) [14]. The combination of these components produces lethal cytotoxic agents [e.g., singlet oxygen (^1^O_2_) and/or other reactive oxygen species] that are responsible for the destruction of malignant cells [4,13,15,16] An obvious advantage of this therapeutic approach is that it is minimally invasive and consequently with great significance for improving patients’ outcomes with either benign or malignant diseases [17]. The specificity of PDT relies on the preferential accumulation of the PS in the diseased tissue and on the localized light delivery, so the damaging should be confined to the irradiated area [14,18,19]. It can be applied before or after chemotherapy, ionizing radiation, or surgery, without compromising these treatments [20].

In recent years, several groups have been dedicated to the optimization of PDT by exploring methodologies that induce structural modifications on the porphyrinic macrocycle core, aiming to reach compounds with adequate photophysical and hydrophobic/hydrophilic properties and with high specificity for the tumor [13,17,21,22]. In fact, PDT efficiency can be enhanced by improving intracellular targeting ability of a PS [17]. One of the approaches to reach this selectivity is based on the synthesis of porphyrins bearing targeting moieties, like carbohydrate units [13,15,19,23,24,25,26], cyclodextrins [13,27,28,29,30], human serum albumin and monoclonal antibodies [13,31,32,33], among others. Other moieties that merit attention are based on triazoles. These heterocycles play an important role in organic and in medicinal chemistry due to their easy synthetic access accompanied by a wide range of biological activities [34]. They have high aromatic stabilization and their capacity of hydrogen bonding could be an advantage in the binding to biomolecular targets [35]. Actually, derivatives with triazolyl substituents demonstrated marked anticancer effect on several malignant tumor cell lines [36,37,38,39,40]. Triazoles have been also used to functionalize porphyrin derivatives, mainly by the “click chemistry” approach [41,42]. This copper(I) catalyzed azide-alkyne cycloaddition has been applied in the derivatization of several tetrapyrrolic molecules such as porphyrins, phthalocyanines, and corroles [41] and many studies have been conducted on the use of these “click-made” porphyrins as polymers [43], in biochemistry [18,24], in materials chemistry [44], and for the preparation of PSs for PDT [45,46,47,48]. However, in such studies a systematic evaluation of the role of the triazole moiety on the PDT efficacy of porphyrins bearing this type of units has not yet been performed.

This work describes an efficient synthetic procedure giving access to a series of β-substituted triazole-porphyrin derivatives (TZ-PORs) and of their PDT efficacy after incorporation into polyvinylpyrrolidone (PVP) micelles. Cellular internalization and phototoxic properties of the PVP-TZ-POR have been compared to those obtained with PVP-TPP in HT-1736 bladder cancer cells and ARPE-19 control cells.

## 2. Results and Discussion

### 2.1. Synthesis and Characterization of New β-Substituted Triazole-porphyrin Derivatives (TZ-PORs)

**Scheme 1:**
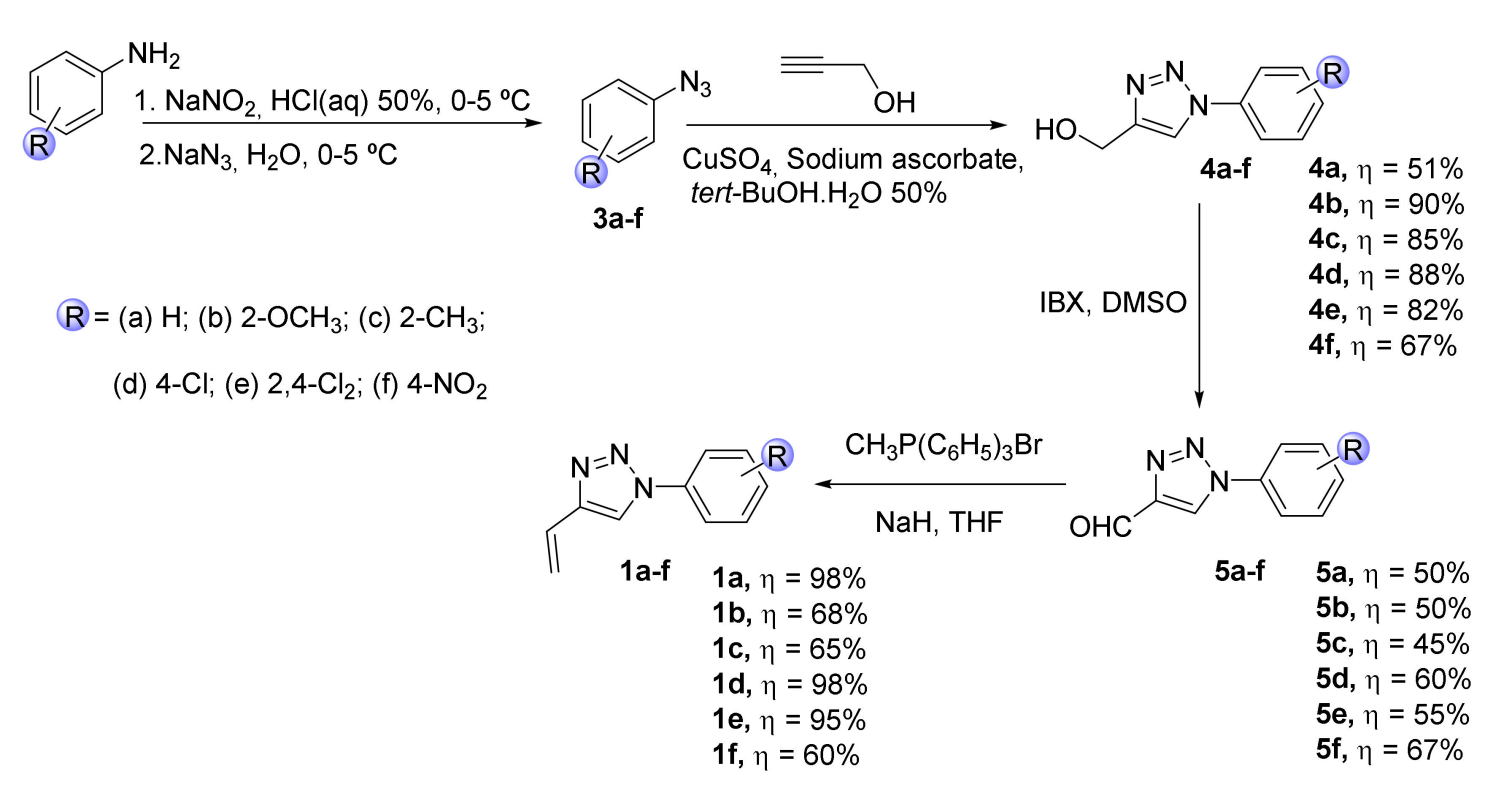
Synthesis of *N*-substituted 4-vinyl-1,2,3-triazoles **1a**–**f**.

In the synthetic access to the required β-substituted triazole-porphyrin derivatives (TZ-PORs) **7a**–**f** under Mizoroki-Heck conditions, the *N*-substituted 4-vinyl-1,2,3-triazoles **1a-f** (Scheme 1) were selected as the alkene components and 2-bromo-5,10,15,20-tetraphenylporphyrinatozinc(II) (**2**) as the aryl halide partner.

The alkene partners **1a**–**f** with the phenyl group with no substituent or with electron donating or releasing groups, were prepared by following the methodology described in Scheme 1 [49]. The strategy involved the initial preparation of the aromatic azides **3a**–**f**, by reaction of sodium azide with the diazonium salts generated from the adequate aromatic amines. Then, a Cu(I) catalyzed azide-alkyne cycloaddition (CuAAC) reaction with propargylic alcohol, afforded triazoles **4a**–**f**. Oxidation of the hydroxyl group in these intermediates with 2-iodoxybenzoic acid (IBX) in DMSO allowed the isolation of aldehydes **5a**–**f**; the latter compounds, after reaction with the ylide generated from methyltriphenylphosphonium bromide, afforded the desired 4-vinyl-1*H*-1,2,3-triazoles **1a**–**f**.

**Scheme 2:**
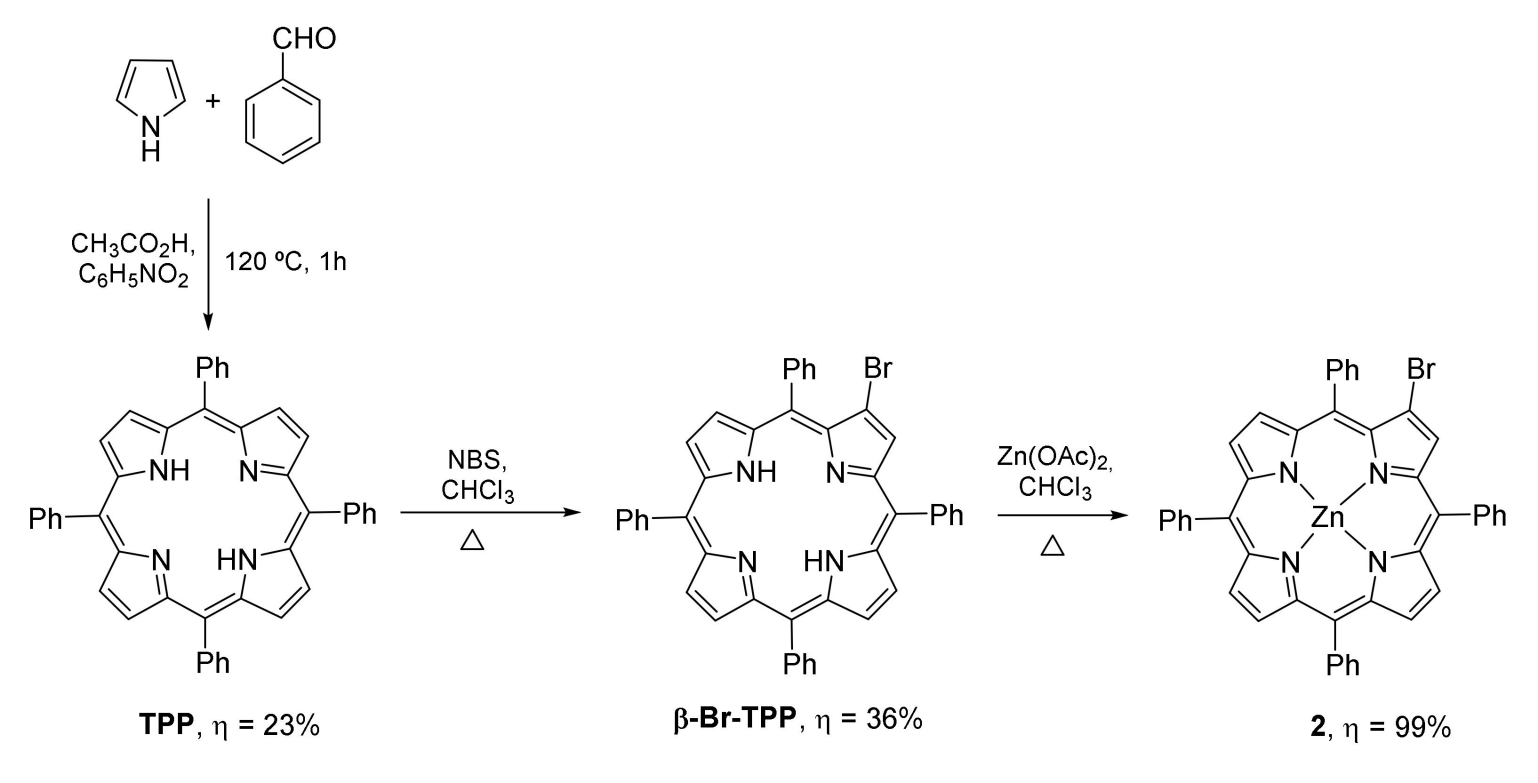
Synthesis of porphyrin **2** used as template in the reactions with the vinyltriazoles.

The porphyrin component was prepared according to literature data by a controlled bromination of 5,10,15,20-tetraphenylporphyrin (**TPP**) with *N*-bromosuccinimide (NBS), affording first 2-bromo-5,10,15,20-tetraphenylporphyrin (**β-Br-TPP**) and after metalation with Zn(OAc)_2_ the 2-bromo-5,10,15,20-tetraphenylporphyrinatozinc(II) (**2**) (Scheme 2) [50,51].

**Scheme 3:**
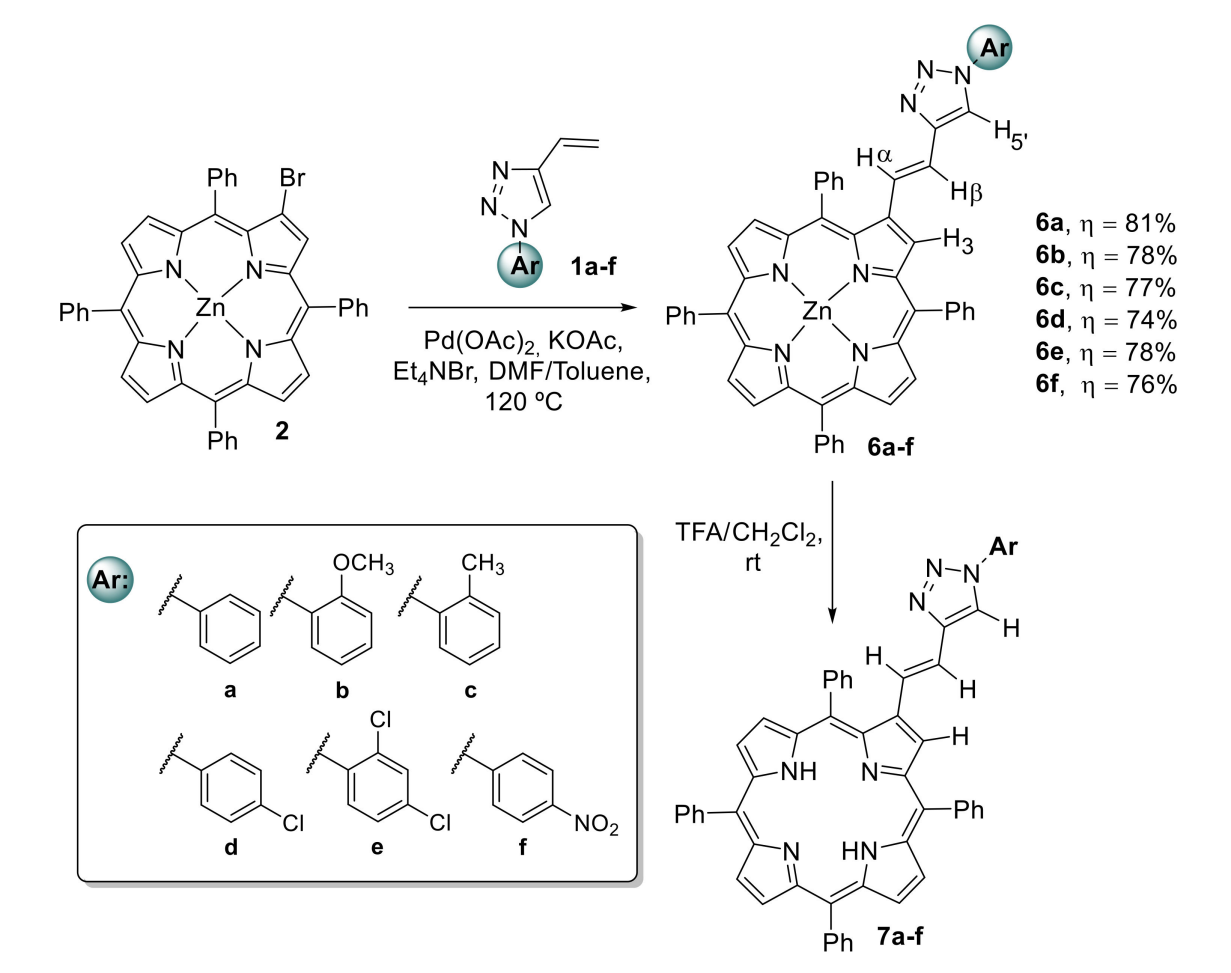
Synthetic route for the synthesis of new β-substituted triazole-porphyrin derivatives (TZ-PORs) **6a**–**f** and **7a**–**f.**

The Heck coupling reactions between bromo-porphyrin **2** and the 4-vinyl-1,2,3-triazoles **1a**–**f** (2 equivalents) were performed in the presence of Pd(OAc)_2_ (20% mol), KOAc (1 equivalent) and Et_4_NBr (1 equivalent) as the catalytic system and using toluene/DMF (2:1) as solvent (Scheme 3) [52]. The 4-vinyl-1,2,3-triazoles were used in slight excess in order to minimize the debromination of porphyrin **2**; this is a side reaction well known in palladium-catalyzed cross-coupling reactions involving halogenated porphyrin derivatives [52]. The reactions were maintained under magnetic stirring during 3 h at 120 °C, being confirmed at that time by TLC the total or almost total consumption of the starting porphyrin into a main product. After the workup and purification of the reaction mixture by preparative TLC, it was possible to conclude by a detailed spectroscopic analysis that the major compounds isolated in very good yields ranging from 74% to 81%, were the new β-substituted triazole-porphyrin derivatives (TZ-PORs) **6a**–**f**. Finally, the demetallation of these complexes with TFA at room temperature allowed to obtain quantitatively the corresponding free-bases **7a**–**f** (Scheme 3).

The structural assignments of all TZ-POR derivatives **6a**–**f** were based on their ^1^H, and ^13^C NMR spectra, and their molecular formulas were confirmed by HRMS (ESI^+^). 2D NMR spectra (COSY, HMBC and NOESY) were also obtained to unequivocally identify the proton and carbon resonances.

The ^1^H NMR spectra of TZ-POR **6a**–**f** are consistent with mono β-substituted structures with the resonance of the β-pyrrolic proton H-3 appearing as a doublet (*J* ~ 0.9 Hz) at δ between 9.04 and 9.13 ppm (see experimental part and Supporting Information). The other β-pyrrolic proton resonances, appearing as a multiplet at δ 8.90–8.91 ppm, were assigned to H-7,8 and H-17,18, and the AB system (*J* ~ 4.7 Hz) at δ 8.80 and 8.90 ppm to the resonances of H-12,13. The resonances of the vinyl protons Hα and Hβ appear as two doublets (*J* ~ 16.0 Hz) at ca. δ 6.80 and 7.30 ppm. COSY correlations were fundamental to unequivocally assign the resonance of Hα and Hβ and also to justify the small coupling constant observed for the β-pyrrolic proton H-3; the long-distance correlation observed between H-3 and the doublet at lower δ allowed to identify it as being Hα. In fact, for derivative **6a** it is possible to observe that Hα appears as a double doublet (*J* = 16.2 and 0.9 Hz) at 6.92 ppm. The value of the coupling constant (*J* ≈ 16.2 Hz) between Hα and Hβ confirms the *trans* configuration of these systems. The resonances due to the protons H5’ of the triazole units were assigned to the singlets that appear at δ ~7.8 ppm; in the case of derivatives **6a**, **6c** and **6d**, NOESY correlations allowed the identification of the resonance of H5’, through correlation with Hβ, that appears under the multiplet related to H-*m,p*-Ph-5,10,15,20. The several multiplets observed also in the more protected aromatic region, were assigned to the resonances of the protons due to the *meso*-phenyl substituents of the porphyrinic macrocycle and of the triazole moieties aryl groups.

The HRMS (ESI^+^) spectra of TZ-POR derivatives **6a**–**f** showed the corresponding molecular ion (M^+·^), confirming their molecular formula.

The structures of the free-base derivatives **7a**–**f** were confirmed by considering their ^1^H NMR, UV-Vis spectra and HRMS analysis. In their ^1^H NMR spectra the characteristic signal at δ ~ – 2.3 ppm due to resonances of the inner *N*-H protons confirmed the success of the demetalation process. As expected, there are no significant differences between the lower-field region of the ^1^H NMR spectra of TZ-POR derivatives **7a**–**f** and of the corresponding zinc(II) complexes.

### 2.2. Incorporation of β-Substituted Triazole-Porphyrins **7a–f** into PVP Micelles

Considering that for an efficient PDT treatment, it is essential to minimize the formation of inactive aggregates in aqueous environment, the TZ-POR derivatives **7a**–**f** were incorporated into PVP micelles. PVP is a water soluble and non-toxic polymer, widely used to modify the compound water solubility, the pharmacokinetics and pharmacological (including antitumor) activity of various biologically active compounds [53]. PVP has been successfully applied as carrier for chlorin e6 (Ce6) and a Ce6-PVP formulation called Photolon has been already approved for PDT [54,55]. This formulation has been extensively studied with respect to its biodistribution and pharmacokinetics, [56,57] intracellular localization [58], interactions with lipoproteins [59], and possible cell death mechanisms [54,55]. Due to its potentiality, PVP has been used with success to prepare formulations of other PSs like phthalocyanines and porphyrins [60,61].

**Scheme 4:**
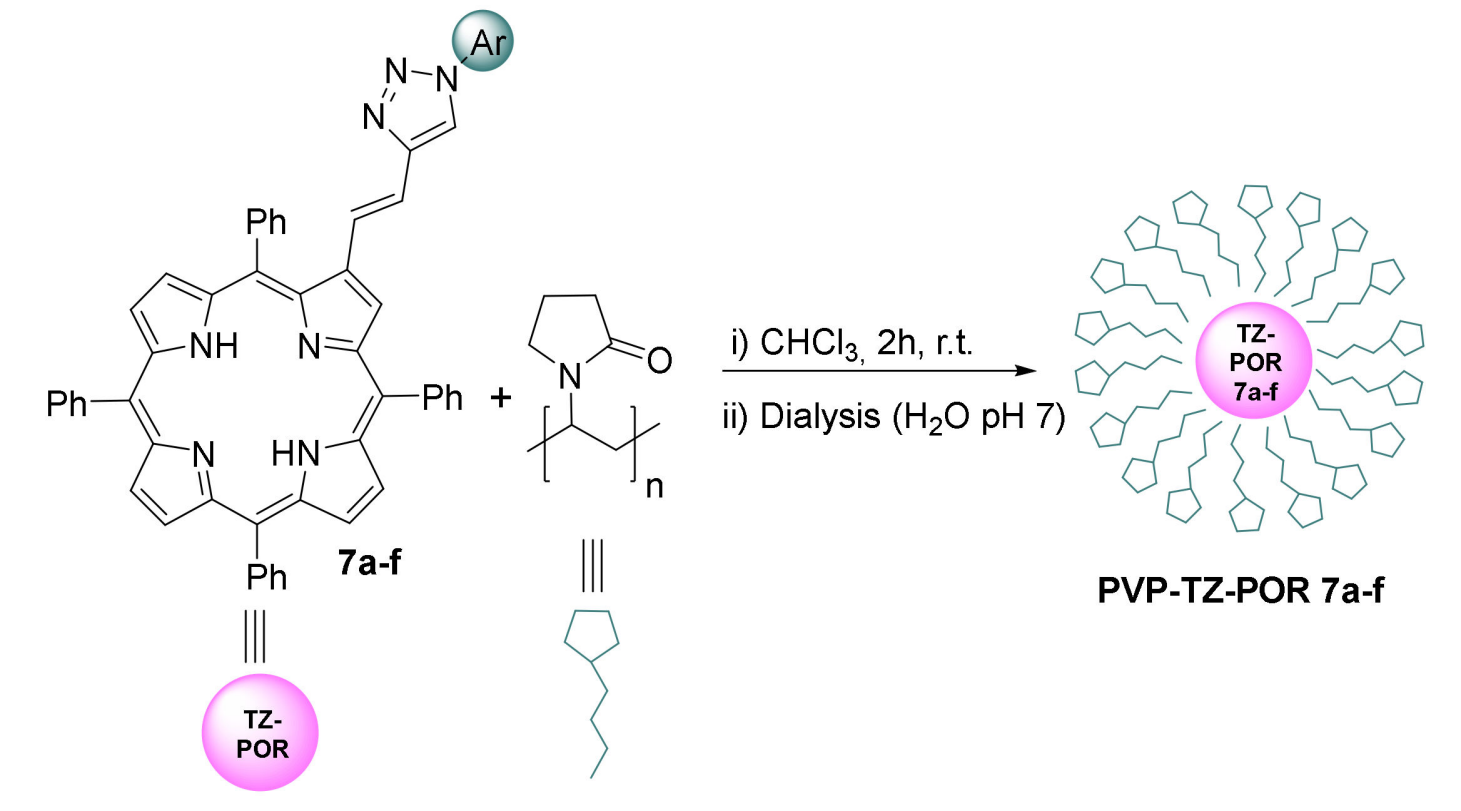
Schematic representation of the incorporation of TZ-POR derivatives **7a**–**f** into PVP micelles.

The incorporation of TZ-POR derivatives **7a**–**f** into PVP was performed according to the literature (Scheme 4) [60,61]. Both components were dissolved in chloroform [ratio PVP (10): **7a**–**f** (1) w/w] and were maintained under stirring for 2 h at room temperature. Then, the organic solvent was removed under a nitrogen atmosphere and then dialysis in distilled water at pH 7 took place. After this process all the porphyrin derivatives afforded stable PVP-TZ-POR **7a**–**f** formulations and no leaching phenomenon of the porphyrin from the micelles was noticed.

### 2.3. Photophysical Characterization of TZ-POR **7a–f** and Formulations PVP-TZ-POR **7a–f**

**Figure 1:**
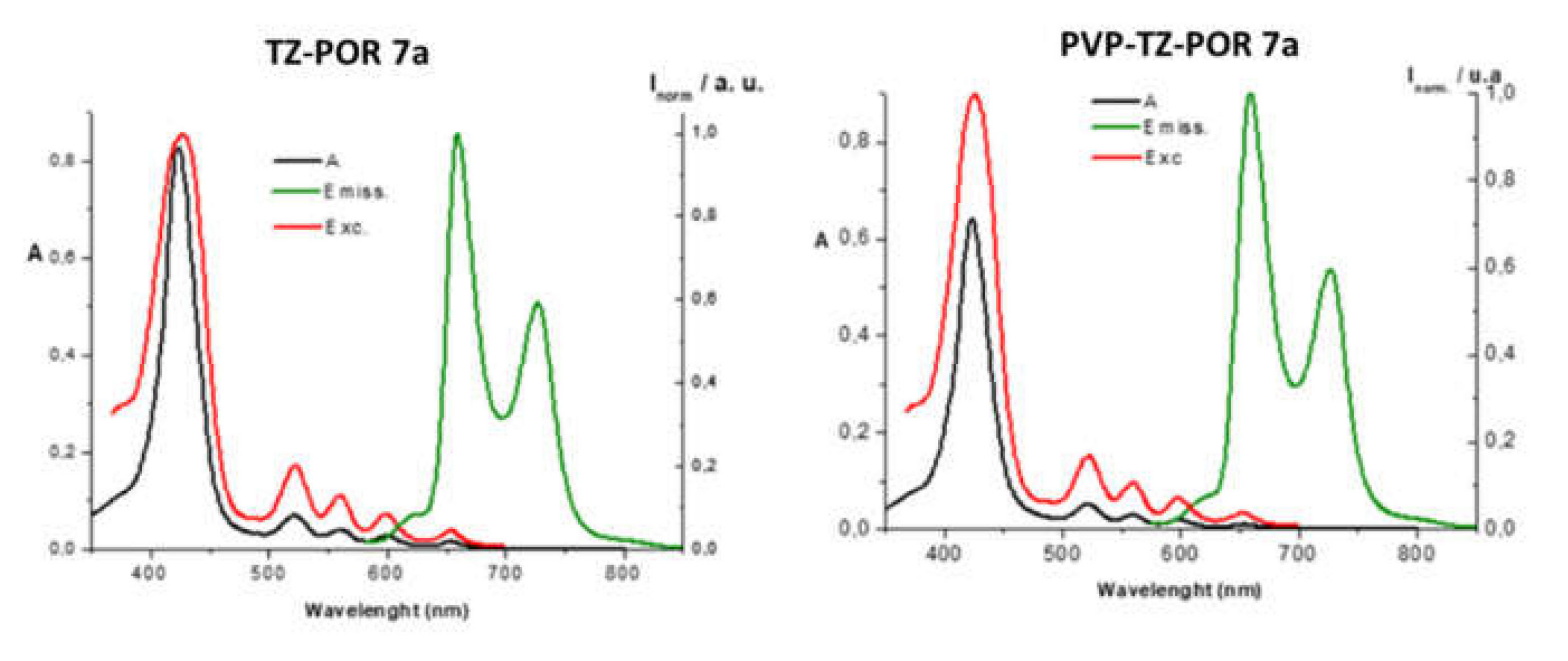
Absorption (A) and normalized emission (Emiss.) and excitation (Exc.) spectra of TZ-POR **7a** *vs* PVP-TZ-POR **7a** in DMF/H_2_O (9:1) ([TZ-POR **7a**] = [PVP-TZ-POR **7a**] = 0.5x10^–6^ M, λ_exc_POR **7a** = 561 nm and λ_emis_ POR **7a** = 728 nm; λ_exc_PVP-TZ-POR **7a** = 560 nm and λ_emiss_PVP-TZ-POR **7a** = 726 nm).

The photophysical characterization of TZ-POR **7a**–**f** and of their formulations PVP-TZ-POR **7a**–**f** was performed in a mixture of DMF/H_2_O (9:1) at room temperature and the main photophysical data are summarized in Table S1 (in the Supplementary Materials). As an example, Figure 1 shows the absorption, excitation, and emission spectra of TZ-POR **7a** before and after its incorporation into PVP.

The absorption spectra of the TZ-POR derivatives **7a**–**f** and of their respective PVP formulations are similar, showing the typical features of free-base porphyrins due to π–π* transitions; the highly intense Soret bands (due to the allowed S0 → S2 transition) appear at 422–424 nm and the four Q bands (due to the S0 → S1 transition) between *ca* 521 and 654 nm. The match between the absorption and the excitation spectra rules out the presence of any emissive impurity. It is worth to emphasize that the PVP-TZ-POR **7a**–**f** formulations in DMF/H_2_O (9:1) follow the Lambert–Beer law, suggesting that the solubility of these compounds is not affected at concentrations up to 30 μM. The fluorescence emission spectra of the triazole derivatives and of their formulations obtained after excitation at approximately 550 nm, also show the same profile, two emission bands centered at ca 650 and 728 nm, which are characteristic of free base porphyrin derivatives (see Figure 1 for TZ-POR **7a** *versus* PVP-TZ-POR **7a**).

In Table S1 the fluorescence quantum yields (ɸF) of the TZ-POR **7a**–**f** and of their formulations determined by the internal reference method using *meso*-tetraphenylporphyrin (**TPP**) in DMF as the standard ([ɸF] = 0.12) are also summarized [62]. The values obtained, varying between 0.08 and 0.14 for the TZ-POR **7a**–**f** and between 0.04 and 0.11 for their PVP formulations, also show that this important photophysical feature was not strongly affected by the incorporation of the porphyrin derivatives into PVP micelles.

The complete incorporation of TZ-PORs **7a**–**f** into the micelles is confirmed by the absence of aggregation, precipitation, and leaching of the PORs from the formulations in aqueous solutions in combination with the good match between the photophysical parameters of TZ-PORs **7a**–**f** and their respective PVP formulations.

### 2.4. Singlet Oxygen and Photostability Studies of the PVP-TZ-POR **7a–f** Formulations

It is well established that the efficiency of a photosensitizer in PDT is highly related with its ability to generate reactive oxygen species (ROS), namely singlet oxygen (^1^O_2_) and radical species, which induce cell death and tissue destruction [8,63,64].

**Figure 2:**
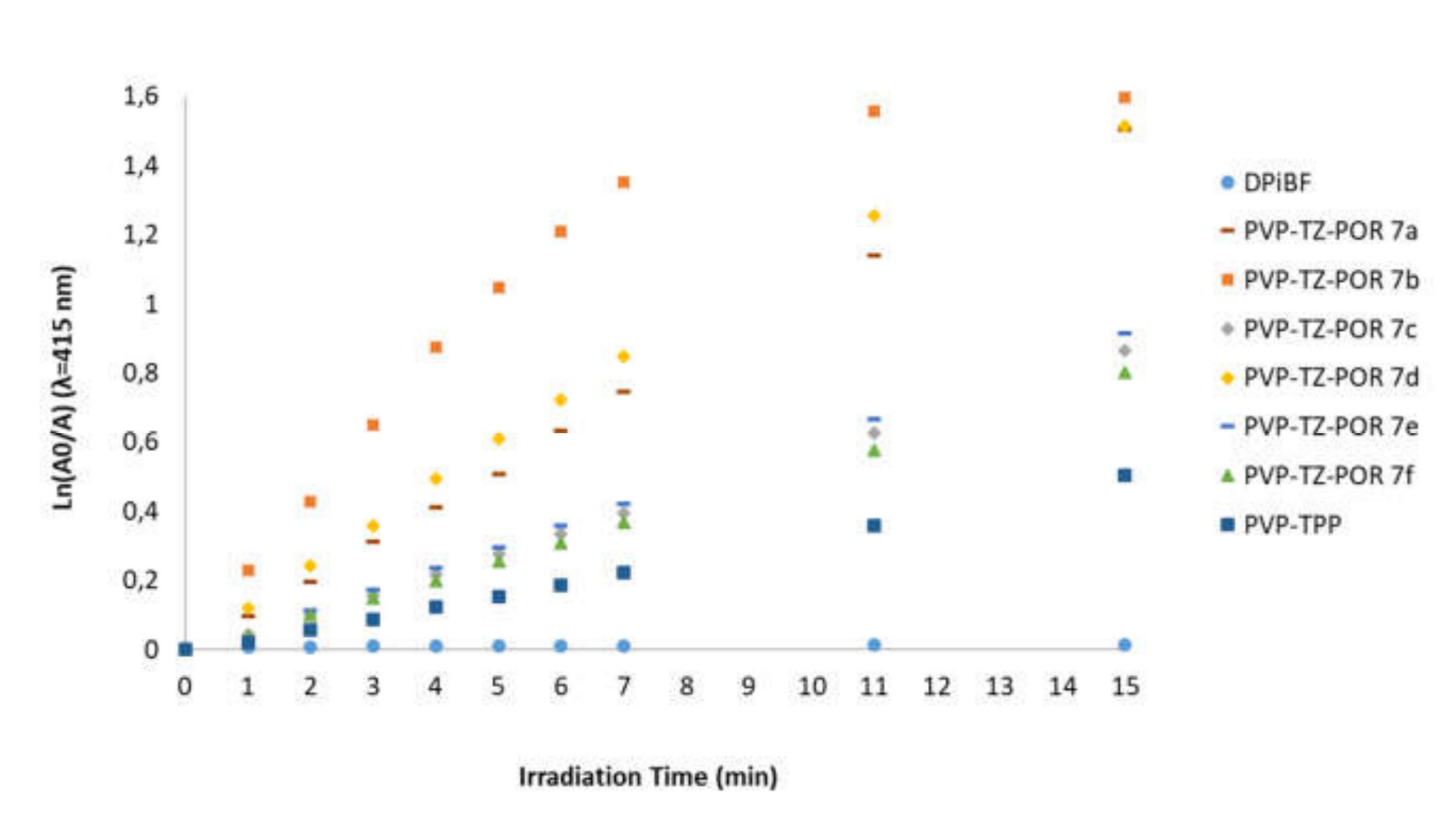
Photodecomposition of DPiBF (50 μM) photosensitized by PVP-TZ-POR **7a**–**f** and PVP-TPP in DMF:H_2_O (9:1) upon irradiation with white light filtered through a cut-off filter for wavelengths < 550 nm, with an irradiance of 20 mW/cm^–2^.

The potential photodynamic effect of PVP-TZ-POR **7a**–**f** was first evaluated by assessing qualitatively their ability to generate ^1^O_2_ using 1,3-diphenylisobenzofuran (DPiBF) as the ^1^O_2_ quencher agent. In this process the ^1^O_2_ produced by the PS reacts with the yellow DPiBF in a [4+2] cycloaddition process, affording the colorless ortho-dibenzoylbenzene. Since DPiBF absorbs at 415 nm, it is possible to follow the capability of the PS to generate ^1^O_2_ by measuring its absorption decay at that wavelength. Hence, aerated solutions of the formulations PVP-TZ-POR **7a**–**f** and DPiBF (100-fold molar excess) in DMF/H_2_O (9:1) were exposed to white light filtered through a cut-off filter for wavelengths < 550 nm, while the absorption of DPiBF at 415 nm was monitored (Figure 2) [65,66,67]. TPP was also incorporated into PVP micelles (PVP-TPP) and used as reference [68,69].

As shown in Figure 2, all formulations are capable to generate ^1^O_2_, since significant photodegradation of DPiBF is observed. It is also evident that all PVP-TZ-POR **7a**–**f** are better ^1^O_2_ generators than the reference PVP-TPP. From these results it is also possible to highlight PVP-TZ-POR **7a**, **7b** and **7d** formulations as the best ^1^O_2_ generators.

The photostability of a PS is also an important requirement for PDT, since this parameter strongly determines the photodynamic efficacy [14,22]. The photostability of PVP-TZ-POR **7a**–**f** formulations was determined in PBS at different irradiation times, by monitoring the absorption Soret band (λ = 420 nm) intensity under white light at a rate of 20 mW.cm^–2^. After 60 min of irradiation, the formulations presented absorption decays between 5 and 12%, which are comparable with PVP-TPP behavior (see Table S2). As such, it is possible to conclude that the derivatization of porphyrin with the triazole units and the incorporation of the resulting macrocycles into the PVP micelles did not compromise their photostability.

### 2.5. In Vitro Studies of the Photodynamic Effect of PVP-TZ-POR **7a–f** in Human Bladder Cancer Cells

The photodynamic effect of PVP-TZ-POR **7a**–**f** was studied in human bladder cancer cells derived from transitional cell carcinoma (HT-1376 cell line). Their PDT efficiency was compared with the results obtained in control ARPE-19 cells (a human retinal pigment epithelial cell line), with the same epithelial origin than HT-1376 cell line.

#### 2.5.1. Cellular Uptake of PVP-TZ-POR **7a–f**

**Figure 3:**
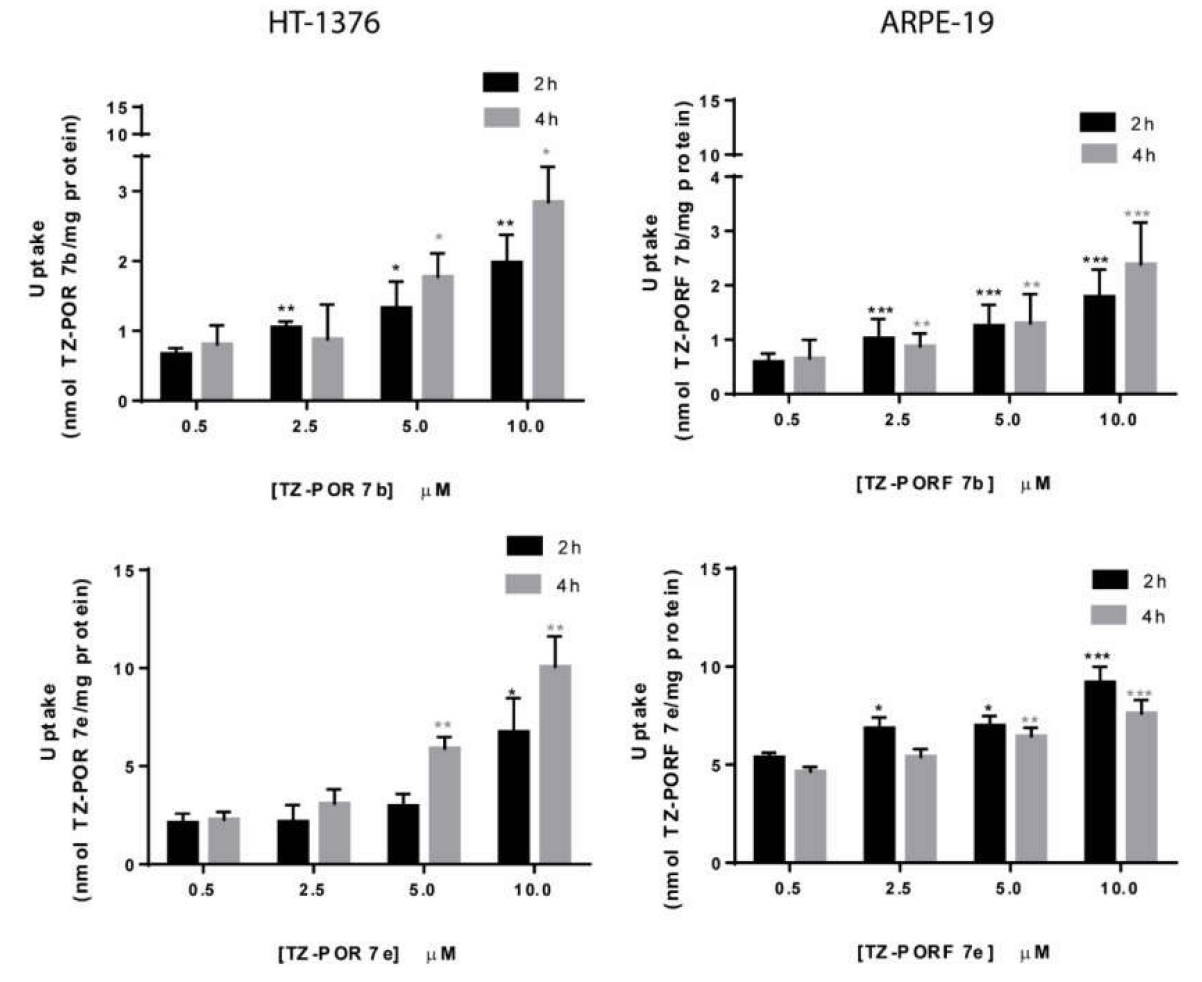
Intracellular uptake of PVP-TZ-POR **7b** and **7e** by HT-1376 and ARPE-19 cells. Data are the mean ± S.D. of at least three independent experiments performed in triplicates: *(*p* < 0.05), **(*p* < 0.01), ***(*p* < 0.001) significantly different from uptake of PSs at lower concentration.

The capacity of PVP-TZ-POR **7a**–**f** to accumulate inside the cancer cells was evaluated by spectrofluorometry, using human bladder cancer cells derived from transitional cell carcinoma (HT-1376 cell line) and the results were compared with those obtained with the control ARPE-19 cell line. To evaluate the influence of the triazole substituent, PVP-TPP formulation was also studied under the same conditions. In these assays, the cells were incubated in the dark with increasing concentrations of each PS micelles (0.5, 2.5, 5, and 10 µM) in PBS for 2 h and 4 h. Figure 3 shows the results obtained for intracellular uptake of PVP-TZ-POR **7b** and **7e** by HT-1376 and ARPE-19 cells. The cellular uptake of the remaining formulations [PVP-TZ-POR **7a**, **7c**, **7d**, **7f** and PVP formulation of triazole free macrocycle (PVP-TPP) is presented in Figure S19). The results indicate that the cellular uptake of all PVP-TZ-POR formulations in HT-1376 cells is concentration and time-dependent, reaching the maximum at 10 µM of PS and after 4 h of incubation in all cases. Particularly PVP-TZ-POR **7b** and the reference PVP-TPP presented the lowest intracellular accumulation, 2.84 ± 0.25 and 3.26 ± 0.23 nmol of PS/mg of protein, respectively, at 10 µM of PS and after 4 h. PVP-TZ-POR **7e** is the formulation with higher intracellular accumulation, with 19.1 ± 2.61 nmol of PS/mg of protein, under the same conditions. However, a different profile for the cellular uptake in ARPE-19 cells was observed. Although ARPE-19 cellular uptake of PVP-TZ-POR **7b** and **7c** was also concentration and time-dependent (please see Figure S19), reaching the maximum at 10µM of PS and after 4 h of incubation, for the other PS-formulations this was not observed. For PVP-TZ-POR **7a**, **7d**, **7f,** and **7d** the maximum intracellular accumulation does not differ between 2 and 4 h of incubation. In what concerns the absolute values of the cellular uptake, they do not differ significantly from the ones observed in the HT-1376 cell line, being PVP-TZ-POR **7b** and **7e** the ones with lower and higher intracellular accumulation, respectively.

**Figure 4:**
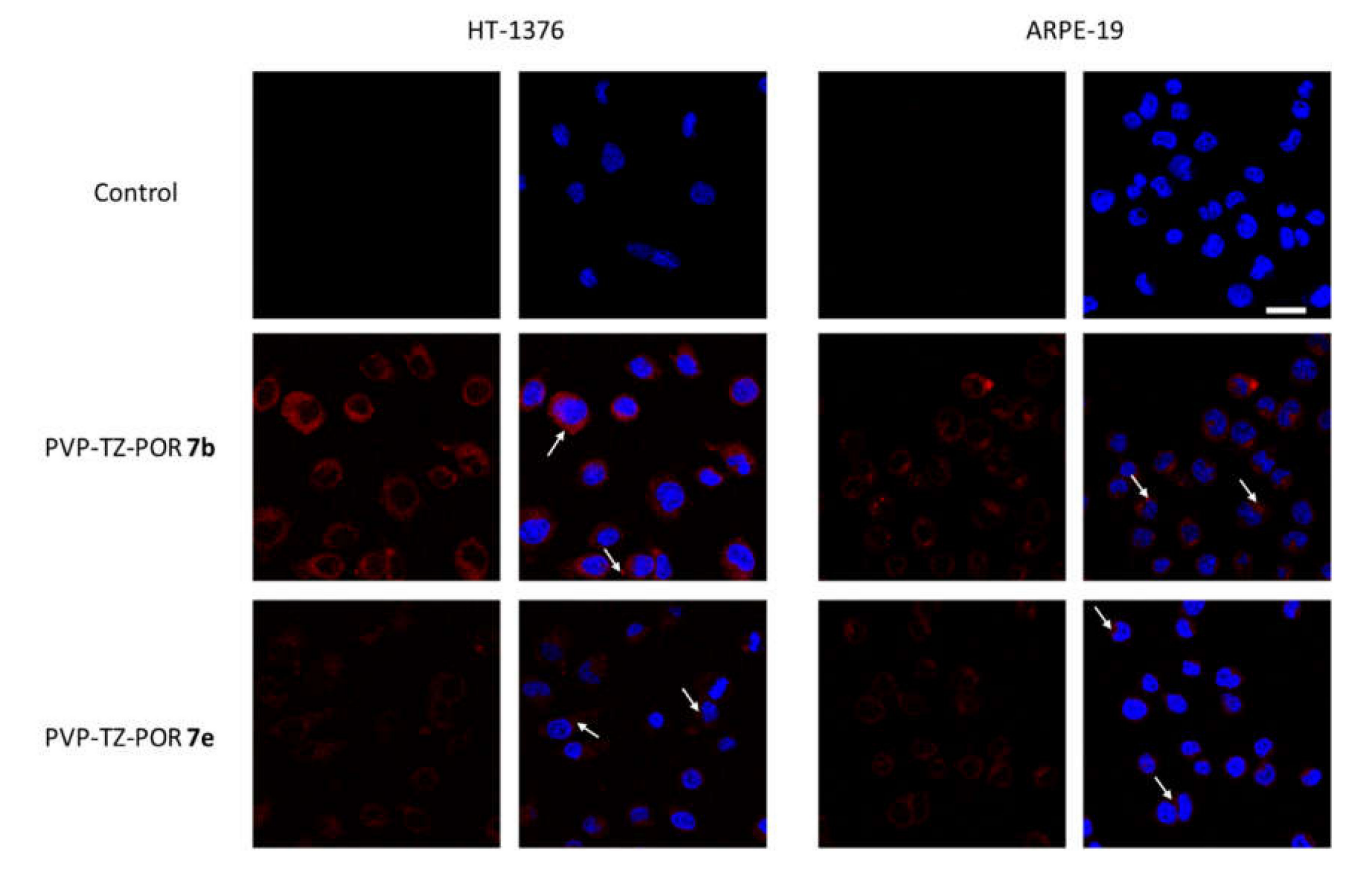
Representative fluorescence images of HT-1376 and ARPE-19 cell lines incubated with 10 µM of PSs PVP-TZ-POR **7b** (red) and **7e** (red) for 4 h in darkness and cell nucleus stained with 4’,6-diamidino-2-phenylindole (DAPI; blue). Scale bar 20 µm.

The spectrofluorometric data was confirmed by confocal microscopy, showing that cells treated with PVP-TZ-POR **7a**–**f** and PVP-TPP formulations for 4 h exhibit fluorescence with occasional strong bright spots in the perinuclear regions (Figure 4, examples for PVP-TZ-POR **7b** and **7e** -white arrows; the remaining PVP-TZ-PORs at Figure S20). It seems that there are no significant differences in the subcellular distribution of most of the formulations between HT-1376 and ARPE-19 cell lines. This indiscriminate internalization by HT-1376 and ARPE-19 cell lines may be related to the composition of the PVP micelles formulation used for the administration of the TZ-POR **7a**–**f** PSs. PVP polymer is known to form pH-sensitive polymeric micelles for extracellular and intracellular drug smart release [70] and it is internalized mediated by endocytosis [71]. These systems are known to release the drug in response to the slightly acidic extracellular fluids of tumor tissue after accumulation via the enhanced permeability and retention effect [71,72]. This fact suggests that interstitial pH in tumor tissue is important to the PS liberation. Thus, the release of the TZ-POR **7a**–**f** PSs content in cytoplasm of cancer cells may be more effective than in non-cancer cells. Moreover, the endosomal and lysosomal pH is lower than the normal physiological pH [71,72], which can also impact the release profile of the compounds from PVP micelle. The release of TZ-PORF **7a**–**f** from the micelle can eventually be different in the two cell lines, leading to a different subcellular localization of the TZ-PORF **7a**–**f** and a distinct viability response pattern.

#### 2.5.2. Cell Viability after PDT Treatment with PVP-TZ-POR **7a–f**

**Figure 5:**
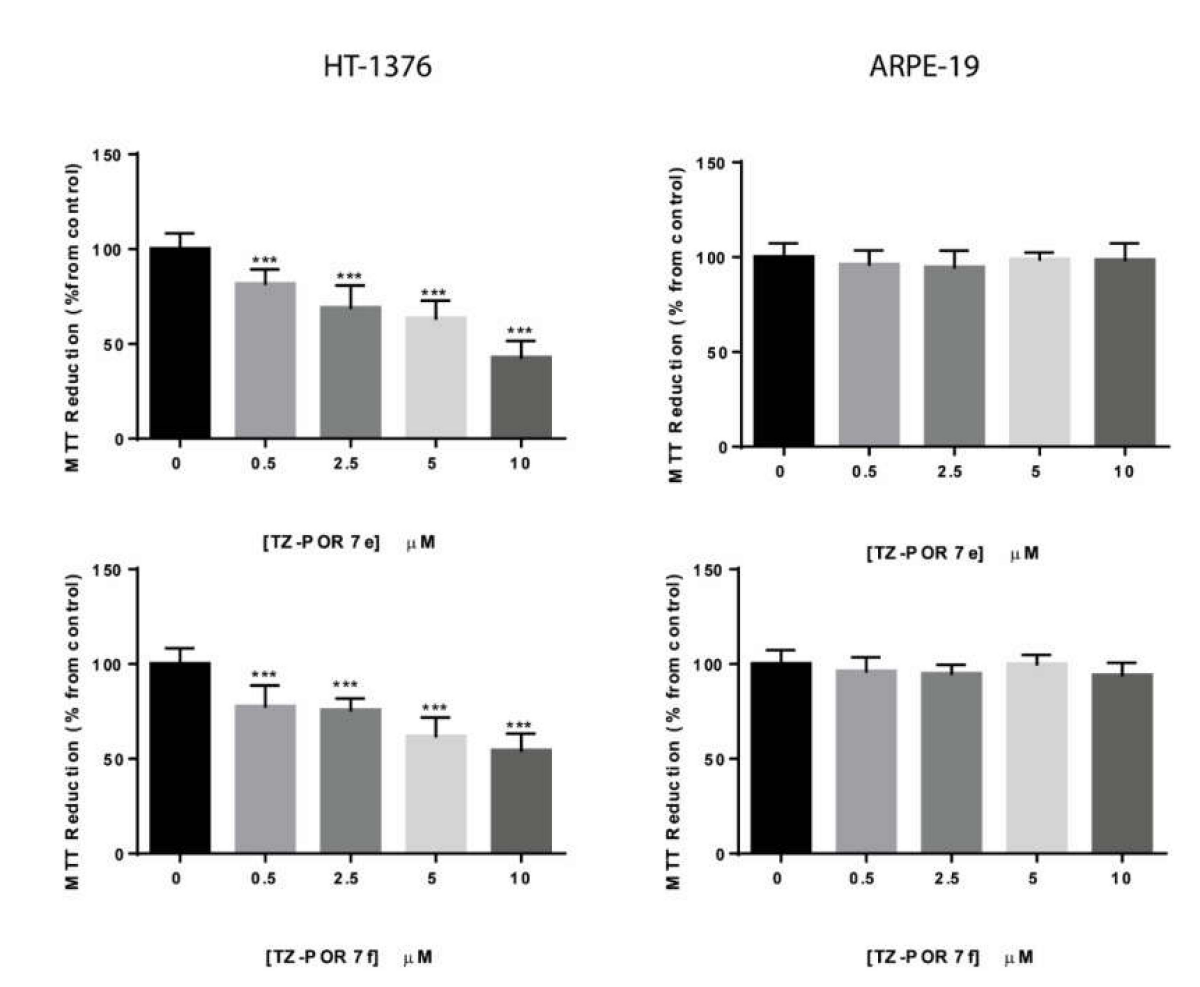
Cell survival of HT-1376 and ARPE-19 cells, incubated in the dark during 4 h with PSs PVP-TZ-POR **7e** and **7f** at different concentrations and irradiated with white light for 40 min with an irradiance of 20 mW.cm^-2^. Data are the mean value ± S.D. of at least three independent experiments performed in triplicates: *(*p* < 0.05), **(*p* < 0.01), ***(*p* < 0.001) significantly different from MTT reduction of the control (%).

The photodynamic effect of the PVP-TZ-POR **7a**–**f** micelles was studied in the bladder cancer cell line HT-1376 and in the control epithelial cell line ARPE-19, at four different concentrations, 0.5, 2.5, 5, and 10 µM. After the incubation with PVP-TZ-POR **7a**–**f** micelles in the dark for 4 h, the cells were irradiated with white light for 40 min with an irradiance of 20 mW.cm^–2^. To evaluate the influence of the triazole substituent, PVP-TPP micelles were also studied. In order to evaluate the toxicity of the PVP polymer, a solution of PVP in PBS at the maximum quantity used in PVP-TZ-POR micelle was also tested. The cell viability was measured using the MTT colorimetric assay after 24 h of PDT protocol. The same protocols without the irradiation procedure were performed to evaluate the cytotoxic effect of all micelles. The results obtained in the cell survival are presented in Figure S21 and Figure S22 and, as an example, Figure 5 presents the results obtained for PVP-TZ-POR **7e** and **7f**.

No cytotoxicity was observed in cells incubated with PVP, PVP-TPP, and PVP-TZ-POR **7a**–**f** in the dark for at least 24 h after treatment. However, when HT-1376 bladder cancer cells were incubated with PVP-TZ-PORF **7a**–**f** and with PVP-TPP micelles and then irradiated, an increase of the phototoxicity was observed, although being dependent on the PS concentration. Derivative TZ-POR **7c** showed to be the most active derivative causing the highest decrease in HT-1376 cells viability of approximately 80 % for the higher concentration. On the opposite side, TZ-POR **7b** is the less active PS on the reduction of HT-1376 cells viability, causing a reduction on that of approximately 30%. This seems to be correlated with its lower accumulation inside HT-1376 cells.

The toxicity induced by PVP-TZ-POR **7a**–**f** formulations in the ARPE-19 cell line is, in general, significantly lower than the one observed in HT-1376 cells. Table 1 summarizes the results obtained for the cell survival of HT-1376 and ARPE-19 cells incubated in the dark for 4 h with 10 µM of each PS PVP-TZ-POR **7a**–**f** and PVP-TPP, prior to PDT. Derivatives TZ-POR **7a**, **7b**, **7c,** and **7d** affect the viability of ARPE-19 cells, after the PDT treatment, only at the highest concentration (Table 1, entries 2, 3, and 4 and Figure S21). Interestingly, derivatives TZ-POR **7e** and **7f** were efficiently internalized by ARPE-19 cells, however under photoactivation they did not induce cell death, at least for the used concentrations (Figure 5 and Table 1, entries 5 and 6). These results clearly indicate that these PSs have a significant effect on the HT-1376 cells viability, without significantly affecting ARPE-19 cell line viability. These results suggest that the subcellular distribution of the TZ-POR **7a**–**f** after micelles release could be different in both cell lines, leading to different results in cell viability. Further studies are needed to understand the molecular mechanisms underlying the cytotoxic effects after photoactivation in cancer cells.

Additionally, it is possible to observe that the photodynamic effect of PVP-TPP formulation on control ARPE-19 cells is similar or even more significant than the one observed for HT-1376 cells, mainly when the higher concentration was tested (Table 1, entry 7). This fact shows the massive importance of the triazole unit on the cytotoxic effect of these PSs for the tumoral cell line. As expected, PVP itself is nontoxic for both normal and cancer cells after PDT treatment.

## 3. Materials and Methods

### 3.1. Synthesis and Characterization of New β-Substituted Triazole-porphyrin Derivatives (TZ-PORs)

^1^H and ^13^C NMR spectra were recorded on a Bruker Avance 300 spectrometer at 300.13 and 75.47 MHz and on a Bruker Avance 500 spectrometer at 500.12 and 125.77 MHz, respectively. CDCl_3_ was used as solvent and tetramethylsilane (TMS) as internal reference. Chemical shifts are expressed in δ (ppm) and the coupling constants (*J*) are expressed in Hertz. HRMS were recorded on a VG AutoSpec M mass spectrometer using CHCl_3_ as solvent and 3-nitrobenzyl alcohol (NBA) as matrix. The UV–Vis spectra were recorded on an UV-2501 PC Shimadzu spectrophotometer using CHCl_3_ as solvent. Fluorescence emission spectra were recorded on a Horiba Jobin-Yvon Fluoromax 3 spectrofluorometer and fluorescence quantum yields of compounds TZ-POR **7a**–**f** and PVP-TZ-POR **7a**–**f** were measured by using a solution of **TPP** in DMF as a standard ([ɸ] = 0.12); all values were corrected by taking into account the solvent refraction index. Flash chromatography was carried out using silica gel (230–400 mesh) and preparative thin-layer chromatography was carried out on 20 × 20 cm glass plates coated with silica gel (1 mm thick). The reactions were routinely monitored by thin layer chromatography (TLC) on silica gel precoated F254 Merck plates.

#### 3.1.1. General Procedure for 4-Vinyl-1,2,3-triazoles **1a–f**

The 4-vinyl-1,2,3-triazoles **1a**–**f** were synthesized according to a previously reported method [49]. The 1,2,3-triazoles **4a**–**f** were obtained from Cu(I)-catalyzed Huisgen 1,3-dipolar cycloaddition reactions between aromatic azides **3a**–**e** (obtained from diazotization of the corresponding anilines with NaNO_2_/HCl, followed by the reaction with NaN_3_) and propargylic alcohol. Then, 4-carboxaldehyde-1H-1,2,3-triazoles (**5a**–**f**) were prepared by oxidation of the alcohols (**4a**–**f**) using 2-iodoxybenzoic acid (IBX), and the 4-vinyl-1H-1,2,3-triazoles **1a**–**f** were obtained by a Wittig reaction starting from aldehydes **5a**–**f**.

#### 3.1.2. Synthesis of 2-Bromo-5,10,15,20-tetraphenylporphyrinatozinc(II) (**2**)

In a round 3-neck flask 120 mL of glacial acetic acid, 70 mL of nitrobenzene and benzaldehyde (5.9 mL, 57.7 mmol, 1 equiv) were added and this mixture was maintained at 120°C for 15 min under magnetic stirring. Then, pyrrole (4 mL, 57.7 mmol) was added dropwise and the reaction was maintained for 1 h at 120 °C under magnetic stirring. After this time, the reaction mixture was cooled to room temperature. The 5,10,15,20-tetrafenylporphyrin (**TPP**) was obtained by filtration under reduced pressure, directly from the reaction medium after addition of methanol. After recrystallization using a mixture of dichloromethane/methanol, the product was obtained pure in 23% yield (2.015 g, 3.258 mmol) [50]. Then, a mixture of **TPP** (200 mg, 0.33 mmol) and *N*-bromosuccinimide (102 mg, 0.58 mmol) in chloroform (60 mL) was stirred and maintained at reflux for one hour. After this, the reaction mixture was washed with a solution of sodium hydrogen carbonate (100 mL) and the organic phase extracted with chloroform (100 mL) and dried in anhydrous sodium sulfate. The reaction mixture was purified by column chromatography of silica using toluene/hexane (2:1) as eluent. After crystallization with dichloromethane/methanol, 2-bromo-5,10,15,20-tetraphenylporphyrin (**β-Br-TPP**) was obtained in 36% yield (80.3 mg) [51]. The zinc(II) complex of **β-Br-TPP** was obtained by adding zinc(II) acetate (40.5 mg, 0.185 mmol) dissolved in methanol (1 mL) to a solution of **β-Br-TPP** (80 mg, 0.115 mmol) dissolved in 30 mL of chloroform. The mixture was kept under stirring at 60 °C for 30 min. After this time, the reaction mixture was evaporated to dryness and the residue was dissolved in 50 mL of chloroform and washed with distilled water (50 mL) in order to remove the excess of zinc acetate. Then, the organic phase was separated, dried over anhydrous sodium sulfate and evaporated to dryness. The porphyrinic zinc complex **2** was obtained quantitatively after crystallization in chloroform/methanol [51].

#### 3.1.3. General Procedure for the Heck Coupling Reactions of 2-Bromo-5,10,15,20-tetraphenylporphyrinatozinc(II) (**2**) and 4-Vinyl-1,2,3-triazoles **1a–f**

In a two neck round-bottom flask under a nitrogen atmosphere, 2-bromo-5,10,15,20-tetraphenylporphyrinatozinc(II) (**2**, 20 mg, 26.4 μmol) and the 4-vinyl-1,2,3-triazoles **1a**–**f** (2 equiv., 52.8 μmol) were placed. These solids were maintained under nitrogen flux for 5 min. Then, Pd(OAc)_2_ (20% mol %, 5.28 μmol, 1.2 mg), KOAc (2 equiv., 52.8 μmol, 5.18 mg) and Et_4_NBr (2 equiv., 52.8 μmol, 11.1 mg) were added and each mixture was dissolved in toluene (1 mL) and DMF (0.5 mL). The reactions were maintained at 120 °C for 4 h. After cooling, the reactions were quenched with water, extracted with toluene and the organic layers were washed with water, dried over Na_2_SO_4_ and concentrated under reduced pressure. The crude mixtures were purified by preparative TLC, using hexane/CH_2_Cl_2_ (1:1) as eluent.

**TZ-POR 6a**: **^1^H NMR** (500 MHz, CDCl_3_) δ 9.04 (d, *J* = 0.9 Hz, 1H, H-3), 8.86–8.82 (m, 4H, β-H), 8.80 and 8.75 (AB, *J* = 4.6 Hz, 2H, H-12,13), 8.23–8.17 (m, 8H, H-*o*-Ph-5,10,15,20), 7.85–7.72 (m, 15H, H-*m,p*-Ph-5,10,15,20 + H-*o,p*-Ph + H-5’), 7.65 (d, *J* = 8.8 Hz, 2H, H-*m*-Ph), 7.32 (d, *J* = 16.2 Hz, 1H, H-β), 6.92 (dd, *J* = 16.2, 0.9 Hz, 1H, H-α). **^13^C NMR** (75 MHz, CDCl_3_) δ 150.7, 150.6, 150.5, 150.47, 150.42, 150.3, 150.2, 148.6, 143.4, 143.3, 143.0, 142.8, 142.7, 142.3, 134.4, 132.2, 132.1, 131.6, 131.5, 130.4, 130.0, 129.8, 128.7, 128.4, 127.7, 127.5, 126.98, 126.91, 126.6, 126.5, 121.5, 121.48, 121.45, 121.3, 121.1, 120.8, 120.1, 117.2. **UV-Vis** (CHCl_3_): λmax (log ε) = 428 (5.54), 516 (3.75), 555 (4.40), 589 (5.04) nm. **HRMS (ESI^+^):** *m/z* 845.2264. Calcd. for C_54_H_35_N_7_Zn M^+·^ 845.2245.

**TZ-POR 6b**: **^1^H NMR ^1^H NMR** (300 MHz, CDCl_3_) δ 9.10 (d, *J* = 0.7 Hz, 1H, H-3), 8.93–8.87 (m, 4H, β-H), 8.88 and 8.81 (AB, *J* = 4.7 Hz, 2H, H-12,13), 8.27–8.13 (m, 8H, H-*o*-Ph-5,10,15,20), 7.83 (s, 1H, H-5’), 7.82–7.64 (m, 13H, H-*m,p*-Ph-5,10,15,20 + H-*o*-Ph-OCH_3_), 7.53–7.44 (m, 1H, H-*p*-Ph-OCH_3_), 7.18 (d, *J* = 7.8 Hz, 2H, H-*m*-Ph-*o*-OCH_3_), 7.13 (d, *J* = 16.3 Hz, 1H, H-β), 6.72 (d, *J* = 16.3 Hz, 1H, H-α), 4.04 (s, 3H, OCH_3_).**^13^C NMR** (126 MHz, CDCl_3_) δ 150.9, 150.7, 150.6, 150.5, 150.3, 150.3, 150.1, 148.7, 146.7, 146.6, 143.2, 142.9, 142.8, 142.9, 142.7, 134.44, 134.41, 134.3, 132.2, 132.1, 132.0, 131.9, 131.5, 130.4, 130.0, 127.7, 127.6, 127.5, 127.4, 126.7, 126.6, 126.57, 126.54, 126.3, 125.4, 121.7, 121.49, 121.41, 121.0, 120.8, 120.7, 118.8, 112.4, 56.1. **UV-vis** (CHCl_3_)**:** λmax (log ε) = 426 (5.53), 513 (3.87), 554 (4.45), 589 (4.04) nm. **HRMS (ESI^+^):** *m/z* 875.*2372*. Calcd. for C_55_H_42_N_7_OZn M^+·^ 875.2251.

**TZ-POR 6c**: **^1^H NMR** (300 MHz, CDCl_3_) δ 9.05 (d, *J* = 0.8 Hz, 1H, H-3), 8.85–8.82 (m, 4H, β-H), 8.81 and 8.75 (AB, *J* = 4.7 Hz, 2H, H-12,13), 8.24–8.17 (m, 8H, H-o-Ph-5,10,15,20), 7.87–7.68 (m, 15H, H-*m,p*-Ph-5,10,15,20 + H-*o*-Ph-*o*-CH_3_ + H-*p*-Ph-*o*-CH_3_ + H-5’), 7.39–7.35 (m, 2H, H-*m*-Ph-*o*-CH_3_), 7.32 (d, *J* = 17.0 Hz, 1H, H-β), 6.90 (d, *J* = 17.0 Hz, 1H, H-α), 1.25 (s, 3H, CH_3_). **^13^C RMN** (75.5 MHz, CDCl_3_+DMSO d_6_): δ 149.9, 149.7, 149.6, 149.5, 149.4, 149.3, 147.8, 147.6,145.7, 145.4, 143.3, 142.8, 142.6, 141.57, 133.9, 131.5 (C-pyrrolic), 131.4 (C-pyrrolic),131.3 (C- pyrrolic), 130.8 (C-3), 129.5, 128.1, 127.0 (C-5’), 126.9 (C-α), 126.7, 126.3, 125.9, 121.7, 121.6, 120.4, 119.9, 119.7, 117.3 (C-*m*-Ph-*o*-CH3), 116.9 (C-*m*-Ph-*o*-CH_3_), 116.5 (C-β), 116.2, 29.0 (CH3). **UV-Vis** (CHCl_3_**):** λmax (log ε) = 428 (5.40), 513 (3.55), 555 (4.24), 589 (4.84) nm. **HRMS (ESI^+^):** *m/z* 859.2432. Calcd. for C_55_H_37_N_7_Zn M^+·^ 859.2402.

**TZ-POR 6d: ^1^H NMR** (300 MHz, CDCl_3_) δ 9.11 (d, *J* = 0.5 Hz, 1H, H-3), 8.95–8.91 (m, 4H, β-H), 8.89 and 8.80 (AB, *J* = 4.7 Hz, 2H, H-12,13), 8.25–8.16 (m, 8H, H-*o*-Ph-5,10,15,20), 7.83–7.68 (m, 15H, H-*m*,*p*-Ph-5,10,15,20 +H-*o*-Ph-*p*-Cl+ H-5’), 7.64–7.56 (m, 2H, H-*m*-Ph-*p*-Cl), 7.12 (d, *J* = 16.3 Hz, 1H, H-β), 6.84 (d, *J* = 16.3 Hz, 1H, H-α). **^13^C NMR** (125.77 MHz, CDCl_3_) δ 150.7, 150.69, 150.60, 150.4, 150.3, 150.2, 148.6, 148.3, 146.4, 143.4, 142.67, 142.66, 142.3, 135.5, 135.4, 134.6, 134.5, 134.45, 134.42, 134.40, 132.4, 132.3 (H-*o*-Ph-5,10,15,20), 132.29, 132.27, 132.18, 132.11, 131.6 (C-pyrrolic),131.6 (C-pyrrolic), 130.9 (C-pyrrolic), 130.4, 130.3, 130.2, 130.1, 128.8 (C-3), 128.5 (C-α), 127.7, 127.6, 127.6, 127.5, 127.1, 126.9, 126.69, 126.62, 126.5, 122.0, 121.5, 121.3, 121.1, 120.9, 120.7, 118.0, 117.37 (C-β), 117.33. **UV-Vis** (CHCl_3_)**:** λmax (log ε) = 428 (4.97), 554 (3.92), 590 (3.61), 619 (3.39) nm. **HRMS (ESI^+^):** *m/z* 879.1889. Calcd. for C_54_H_34_ClN_7_Zn M^+·^ 879.1856.

**TZ-POR 6e: ^1^H NMR** (300 MHz, CDCl_3_) δ 9.11 (d, *J* = 0.6 Hz, 1H, H-3), 8.94 -8.92 (m, 4H, β-H), 8.89 and 8.79 (AB, *J* = 4.7 Hz, 2H, H-12,13), 8.21 -8.18 (m, 8H, H-*o*-Ph-5,10,15,20), 7.90-7.89 (m, 1H, H-*o*-Ph-2,4-Cl_2_), 7.83–7.68 (m, 12H, H-*m,p*-Ph-5,10,15,20), 7.66 (s, 1H, H-5’), 7.60-7.56 (m, 2H, H-*m*-Ph-2,4-Cl_2_), 7.08 (d, *J* = 16.2 Hz, 1H, H-β), 6.86 (d, *J* = 16.2 Hz, 1H, H-α). **^13^C NMR** (125.77 MHz, CDCl_3_) δ 150.8, 150.7, 150.6, 150.5, 150.4, 150.3, 148.9, 148.4, 147.0, 146.3, 143.5, 142.7, 142.6, 142.6, 142.0, 141.0, 134.5, 134.4, 132.3, 132.29, 132.24, 132.1, 131.7, 130.5, 129.3, 127.7, 127.5, 126.9, 126.7, 126.62, 126.60, 125.6, 121.6, 121.2, 120.9, 120.6, 120.0, 117.3, 116.9. **UV-Vis** (CHCl_3_): λmax (log ε) = 428 (4.89), 553 (3.95), 449 (3.54), 618 (3.41) nm. **HRMS (ESI^+^):** *m/z* 916.1718. Calcd. for C_54_H_33_Cl_2_N_7_Zn M^+·^ 916.1727

**TZ-POR 6f: ^1^H NMR** (300 MHz, CDCl_3_) δ 9.13 (s, 1H, H-3), 8.95-8.93 (m, 4H, β-H), 8.90 and 8.81 (AB, *J* = 4.7 Hz, 2H, H-12,13), 8.48 (d, *J* = 9.0 Hz, 2H, H-*o*-Ph-4-NO_2_), 8.24 -8.18 (m, 8H, H-*o*-Ph-5,10,15,20), 7.95 (d, *J* = 9.0 Hz, 2H, H-*m*-Ph-*p*-NO_2_), 7.85–7.67 (m, 13H, H-*m,p*-Ph-5,10,15,20 + H-5´), 7.20 (d, *J* = 16.4 Hz, 1H, H-β), 6.94 (d, *J* = 16.4 Hz, 1H, H-α).**^13^C NMR** (125.77 MHz, CDCl_3_) δ 150.8, 150.7, 150.6, 150.5, 150.4, 150.3, 148.9, 148.4, 147.0, 146.3, 143.5, 142.7, 142.64, 142.63, 142.0, 141.0, 134.5, 134.4, 132.3, 132.29, 132.24, 132.1, 131.7, 130.5, 129.3, 127.7, 127.6, 127.5, 126.9, 126.7, 126.62, 126.60, 125.7, 121.6, 121.2, 120.9, 120.6, 120.0, 117.3, 116.9. **UV-Vis** (CHCl_3_): λmax (log ε) = 428 (4.98), 552 (3.78), 450 (3.50), 618 (3.22) nm. **HRMS (ESI^+^):** *m/z* 446.1144. Calcd. for [C_53_H_36_N_8_O_2_Zn]^2+^ 446.1121.

#### 3.1.4. General Procedure for the Decomplexation of TZ-POR **6a–f** Derivatives

The demetalation of TZ-POR **6a-f** derivatives was made by reaction of 20 mg of each compound (**6a**: 0.0236 mmol; **6b**: 0.0227 mmol; **6c**: 0.0232 mmol; **6d**: 0.0227 mmol; **6e**: 0.0218 mmol and **6f**: 0.0224 mmol) with TFA-CH_2_Cl_2_ (9:1) (3 mL), and being stirred in the dark at room temperature for 30 min. Chloroform (10 mL) and water (20 mL) were then added and each mixture was neutralized with aqueous sodium carbonate and extracted with chloroform (50 mL); the organic phase was then washed with water (50 mL) and dried over Na_2_SO_4_. The solvent was evaporated under reduced pressure to dryness and the residue was crystallized from chloroform/methanol affording, in each case, the new TZ-POR **7a–f**.

**TZ-POR 7a**: ^1^H **NMR** (300 MHz, CDCl_3_) δ 9.04 (s, 1H, H-3), 8.83-8.82 (m, 4H, β-H), 8.78 and 8.73 (AB, J = 4.9 Hz, 2H, H-12,13), 8.24–8.19 (m, 8H, H-o-Ph-5,10,15,20), 7.84 -7.74 (m, 15H, H-m,p-Ph-5,10,15,20 + H-*o*-Ph + H-5’), 7.64 (t, *J* = 7.7 Hz, 2H, H-*m*-Ph), 7.55–7.53 (m, 1H, H-*p*-Ph), 7.44 (d, *J* = 16.3 Hz, 1H, H-β), 6.88 (d, *J* = 16.3 Hz, 1H, H-α), -2.61 (s, 2H, NH). **UV-Vis** (DMF): λmax (log ε) = 424 (5.31), 522 (4.23), 561 (3.99), 599 (3.84), 654 (3.59) nm. **HRMS (ESI^+^):** m/z 784.3191. Calcd. for C_54_H_38_N_7_ [M+H]^+^ 784.3183.

**TZ-POR 7b: ^1^H NMR** (300 MHz, CDCl_3_) δ 9.04 (s, 1H, H-3), 8.85–8.80 (m, 4H, β-H), 8.77 and 8.73 (AB, *J* = 4.8 Hz, AB, J = 4.7 Hz, 2H, H-12,13), 8.26–8.18 (m, 8H, H-*o*-Ph-5,10,15,20), 7.93 (s, 1H, H-5’), 7.90–7.87 (m, 1H, H-*o*-Ph-OCH_3_), 7.84–7.69 (m, 12H, H-*m,p*-Ph-5,10,15,20), 7.54–7.47 (m, 1H, H-*p*-Ph-OCH_3_), 7.48 (d, *J* = 16.8 Hz, 1H, H-β), 7.24–7.15 (m, 2H, H-*m*-Ph-*o*-OCH_3_), 6.80 (d, *J* = 16.8 Hz, 1H, H-α), 4.10 (s, 3H, OCH_3_), -2.61 (s, 2H, NH). **UV-Vis** (DMF): λmax (log ε) = 424 (5.99), 521 (3.96), 561 (3.72), 598 (3.53), 654 (3.26) nm. **HRMS (ESI^+^):** m/z 814.3289. Calcd. for C_54_H_40_ON_7_ [M+H]^+^ 814.3298.

**TZ-POR 7c: ^1^H NMR** (300 MHz, CDCl_3_) δ 9.03 (s, 1H, H-3), 8.80 - 8.82 (m, 4H, H-β), 8.78 and 8.73 (AB, *J* = 4.8 Hz, 2H, H-12,13), 8.26–8.18 (m, 8H, H-o-Ph-5,10,15,20), 7.84–7.73 (m, 14H, H-m,p-Ph-5,10,15,20 + H-*o*-Ph-*o*-CH_3_ + H-*p*-Ph-*o*-CH_3_), 7.70 (s, 1H- H-5’), 7.43 (d, *J* = 16.4 Hz, 1H, H-β), 7.37–7.29 (m, 2H, H-*m*-Ph-*o*-CH_3_), 6.88 (d, *J* = 16.4 Hz, 1H, H-α), 1.25 (s, 3H, CH_3_), -2.62 (s, 2H, NH). **UV-Vis** (DMF): λmax (log ε) = 423 (5.63), 521 (4.53), 560 (4.26), 598 (4.09), 654 (3.82) nm. **HRMS (ESI^+^):** m/z 798.3160. Calcd. for C_55_H_40_N_7_ [M+H]^+^ 798.3340.

**TZ-POR 7d:**^1^H NMR (300 MHz, CDCl_3_) δ 9.03 (s, 1H, H-3), 8.87–8.80 (m, 4H, H-β), 8.78 and 8.73 (AB, *J* = 4.9 Hz, 2H, H-12,13), 8.27–8.17 (m, 8H, H-o-Ph-5,10,15,20), 7.85–7.73 (m, 14H, H-m,p-Ph-5,10,15,20 + H-*o*-Ph-*p*-Cl), 7.71 (s, 1H, H-5´), 7.61 (d, *J* = 8.9 Hz, 2H, H-*m*-Ph-*p*-Cl), 7.42 (d, *J* = 16.3 Hz, 1H, H-β), 6.89 (d, *J* = 16.3 Hz, 1H, H-α), -2.62 (s, 2H, NH). **UV-Vis** (DMF): λmax (log ε) = 424 (5.11), 521 (4.03), 560 (3.76), 598 (3.58), 654 (3.28) nm. **HRMS (ESI^+^):** m/z 818.2809 Calcd. for C_54_H_37_N_7_Cl [M+H]^+^ 818.2793.

**TZ-POR 7e:**^1^H NMR (300 MHz, CDCl_3_) δ 9.03 (s, 1H, H-3), 8.86–8.80 (m, 4H, β-H), 8.79 and 8.72 (d, *J* = 4.9 Hz, 2H, H-12,13), 8.26–8.17 (m, 8H, H-*o*-Ph-5,10,15,20), 7.98–7.97 (m, Hz, 1H, H-*o*-Ph-2,4-Cl_2_), 7.84–7.74 (m, 12H, H-*m,p*-Ph-5,10,15,20), 7.70 (s, 1H, H5’), 7.68–7.67 (m, m, 2H, H-*m*-Ph-2,4-Cl_2_), 7.41 (d, *J* = 16.3 Hz, 1H, H-β), 6.93 (d, *J* = 16.3 Hz, 1H, H-α), -2.61 (s, 2H, NH). **UV-Vis** (DMF): λmax (log ε) = 423 (5.20), 521 (4.12), 560 (3.84), 598 (3.66), 653 (3.36) nm. **HRMS (ESI^+^):** m/z 825.2419 Calcd. for C_54_H_36_N_7_Cl_2_ [M+H]^+^ 852.2404.

**TZ-POR 7f:**^1^H NMR (300 MHz, CDCl_3_) δ 9.04 (s, 1H, H-3), 8.87–8.80 (m, 4H, β-H), 8.80 and 8.73 (AB, *J* = 4.9 Hz, 2H, H-12,13), 8.51 (d, *J* = 9.1 Hz, 2H, H-o-Ph-4-NO_2_), 8.25–8.19 (m, 8H, H-o-Ph-5,10,15,20), 8.02 (d, *J* = 9.1 Hz, 2H, H-m-Ph-p-NO_2_), 7.85–7.74 (m, 13H, Hm,p-Ph-5,10,15,20 + H5´), 7.41 (d, *J* = 16.2 Hz, 1H, H-β), 6.97 (d, *J* = 16.2 Hz, 1H, H-α) -2.62 (s, 2H, NH). **UV-Vis (DMF):** λmax (log ε) = 423 (5.35), 521 (4.30), 560 (4.03), 598 (3.86), 654 (3.57) nm. **HRMS (ESI^+^):** m/z 829.3047 Calcd. for C_54_H_37_O_2_N_8_ [M+H]^+^ 829.3034.

#### 3.1.5. General Procedure for the Incorporation of TZ-POR **7a–f** Derivatives into PVP Micelles

To chloroform solution of PVP (20 mg in 2 mL) each compound TZ-POR **7a**–**f** (*ca*. 2 mg) dissolved in 2 mL of chloroform was added. The resulting solutions were stirred for 2 h at room temperature and then the chloroform was evaporated under a nitrogen atmosphere. After this procedure, all residues were dissolved in 2 mL of water and dialyzed in distilled water at pH 7, in order to remove any organic solvent used in these preparations. The obtained PVP-TZ-POR **7a**–**f** micelles showed high stability in water. This methodology was also used to incorporate **TPP** in PVP affording PVP-TPP micelles.

**PVP-TZ-POR 7a**: UV-Vis (DMF): λmax (log ε) = 424 (5.22), 521 (4.16), 560 (3.90), 598 (3.72), 653 (3.45) nm.

**PVP-TZ-POR 7b**: UV-Vis (DMF): λmax (log ε) = 422 (5.64), 521 (4.59), 560 (4.37), 598 (4.21), 654 (4.04) nm.

**PVP-TZ-POR 7c**: UV-Vis (DMF): λmax (log ε) = 423 (5.12), 521 (4.05), 559 (3.78), 598 (3.61), 653 (3.72) nm.

**PVP-TZ-POR 7d**: UV-Vis (DMF): λmax (log ε) = 424 (5.31), 521 (4.27), 560 (4.00), 598 (3.82), 653 (3.54) nm.

**PVP-TZ-POR 7e**: UV-Vis (DMF): λmax (log ε) = 423 (5.11), 521 (4.09), 559 (3.82), 598 (3.66), 653 (3.41) nm.

**PVP-TZ-POR 7f:** UV-Vis (DMF): λmax (log ε) = 424 (5.36), 521 (4.08), 560 (3.79), 598 (3.62), 652 (3.28) nm.

#### 3.1.6. Singlet Oxygen Generation Studies

A solution of each compound PVP-TZ-POR **7a**–**f** (0.5 µM) and DPiBF (5 µM) in DMF/H_2_O (9:1) was irradiated in a glass cuvette with white light filtered through a cut-off filter for wavelengths < 550 nm, under magnetic stirring. The production of singlet oxygen was evaluated qualitatively through the absorption decay of DPiBF at 415 nm for 10 min at defined intervals. PVP-TPP was also used as reference.

#### 3.1.7. Photostability Assays

For the photostability assays, solutions of PVP-TZ-POR **7a**–**f** and PVP-TPP at 10 µM were freshly prepared in PBS and kept in the dark at room temperature. The irradiation experiments were performed in magnetically stirred cuvette solutions (with 2 mL of sample), over a period of 60 min with white light delivered by an illumination system (LC-122 LumaCare, London) equipped with a halogen/quartz 250 W lamp coupled to the selected interchangeable optic fiber probe (400–800 nm). The light was delivered at a fluence rate of 20 mW.cm^–2^, measured with an energy meter Coherent FieldMaxII-Top combined with a Coherent PowerSens PS19Q energy sensor. The absorbance at 420 nm was determined at 0, 10, 20, 30, 40, 50, and 60 min periods of time after irradiation. The results are presented in percentage calculated by the ratio of residual absorbance at 420 nm at different periods of time and absorbance before irradiation

### 3.2. In Vitro Studies of the Photodynamic Effect of PVP-TZ-POR **7a**–**f** in Human Bladder Cancer Cells

Human bladder cancer cell line HT-1376 derived from high-grade transitional cell carcinoma (from the American Type Culture Collection, ATCC, Manassas, VA, USA) and human retinal pigment epithelial ARPE-19 cells (from the American Type Culture Collection, ATCC, Manassas, VA, USA) were cultured in Roswell Park Memorial Institute medium (RPMI-1640) supplemented with 10% (v/v) of fetal bovine serum (Life Technologies, Carlsbad, CA, USA), 100 U/mL penicillin, 100 mg/mL streptomycin and 0.25 mg/mL amphotericin B (Sigma).

#### 3.2.1. Cellular Uptake of PVP-TZ-POR **7a–f**

After incubation with PVP-TZ-POR **7a**–**f** for 4 h in the dark, HT-1376 and ARPE-19 cells were immediately washed with PBS buffer and lysed in 1 % m/v sodium dodecyl sulfate (SDS; Sigma) in PBS buffer at pH 7.0. PVP-TZ-POR **7a**–**f** intracellular concentration was determined by spectrofluorimetry using a microplate reader Synergy HT, BioTek, Winooski, VT, USA, with excitation and emission wavelengths set at 675 nm. The results were normalized for protein concentration (determined by bicinchoninic acid reagent; Pierce, Rockford, IL, USA).

For microscopic evaluation, HT-1376 and ARPE-19 cells were grown for 24 h on glass coverslips coated with poly-L-lysine (Sigma). The cells were incubated with 10 µM of PVP-TZ-POR **7a**–**f** for 4 h, at 37 °C. After incubation, cells were fixed with 4% paraformaldehyde (PFA; Merck, Darmstadt, Germany) for 10 min at room temperature. The samples were then rinsed in PBS, and mounted in VectaSHIELD mounting medium containing 4’,6-diamidino-2-phenylindole (DAPI; Vector Laboratories, CA, Burlingame) for visualization under a confocal microscope (LSM 510, Carl Zeiss, Gottingen, Germany). For detection of each TZ-POR **7a**–**f**, the specimen was excited at 633 nm and its emitted light was collected between 653–750 nm. For DAPI detection, specimen was excited at 405 nm and its emitted light was collected between 430–500 nm.

#### 3.2.2. Phototoxicity of PVP-TZ-POR **7a–f** Formulations

Cells were seeded (9.4 × 104 cells.cm^–2^) in 96-well cell culture plates and maintained in culture medium under an air atmosphere containing 5% of CO_2_. After seeding the cells overnight, they were washed twice with PBS and incubated in darkness (at 37 °C under air atmosphere containing 5% of CO_2_) with solutions of PVP-TZ-POR **7a**–**f**, PVP-TPP and PVP in PBS. The cells were then washed twice with PBS and covered with 100 µL of fresh medium. Cells were irradiated (using the illumination system referred above for the photostability assays) for 40 min with white light at a fluence rate of 20 mW.cm^–2^. After irradiation, cells were incubated in a humidified incubator in an atmosphere containing 5% of CO_2_ and 95% of air. After 24 h of PDT, cell phototoxicity was determined by the MTT colorimetric assay, using 3-[4,5-dimethylthiazol-2-yl]-2,5-diphenyl-tetrazolium bromide (MTT, yellow, Sigma).

For the dark toxicity evaluation of PVP-TZ-POR **7a**–**f**, the same protocol was applied, however without the irradiation procedure.

#### 3.2.3. MTT Assay

Cell metabolic activity after PVP-TZ-POR **7a**–**f**, PVP-TPP and PVP incubation in the dark, or with irradiation, or with both was determined 24 h after treatments by measuring the ability of cancer cells to reduce 3-[4,5-dimethylthiazol-2-yl]-2,5-diphenyl-tetrazolium bromide (MTT, Sigma), to a colored formazan using a microplate reader (Synergy HT, Biotek, Winooski, VT, USA). The data were expressed in percentage of control (*i.e.,* optical density of formazan from cells not exposed to PSs).

#### 3.2.4. Statistical Analysis

The results are presented as mean of at least 3 independent assays with 3 replicates per assay six. The statistical analysis was performed with GraphPad Prism (GraphPad Software, San Diego, CA, USA). Statistical significance among two conditions was assessed using the nonparametric Mann-Whitney test.

## 4. Conclusions

New β-substituted triazole-porphyrin derivatives **6a**–**f** (TZ-PORs) were efficiently obtained through Heck coupling reaction between 2-bromo-5,10,15,20-tetraphenylporphyrinatozinc(II) (**2**) and 4-vinyl-1,2,3-triazoles **1a**–**f**. For the photodynamic studies, TZ-PORs **6a**–**f** derivatives were demetalated and successfully incorporated into polyvinylpyrrolidone (PVP) micelles, affording stable PVP-TZ-POR **7a**–**f** formulations. The structures of all compounds were confirmed by adequate spectroscopic techniques and the photophysical characterization of TZ-POR derivatives and of their PVP formulations yielded the conclusion that the key features for an efficient photodynamic action was not compromised by the PS incorporation into the micelles. Singlet oxygen generation studies have shown that all formulations PVP-TZ-POR **7a**–**f** are capable to generate this cytotoxic species even better than PVP-TPP used as reference. All formulations presented adequate photostability comparable to PVP-TPP.

The cellular uptake studies of PVP-TZ-POR **7a**–**f** on the cancer HT-1376 cell line have shown that the PSs internalization of PVP-TZ-POR **7a**–**f** is concentration- and time-dependent reaching the maximum at PS concentration 10 µM and in general all the hybrids have higher cellular uptake than the reference PVP-TPP. Nevertheless, a different cellular uptake profile was observed in control ARPE-19 cells, whereupon for most of the formulations the maximum intracellular accumulation does not differ between 2 and 4 h of incubation. The photodynamic effect of PVP-TZ-POR **7a**–**f** formulations on HT-1376 and ARPE-19 cell lines showed to be dependent on the PS concentration, on the presence of the triazole unit as well as on its substituents. In general, in vitro results show that PVP-TZ-POR **7a**–**f** are effective against bladder cancer cells after PDT. These results also show that PVP-TZ-POR **7e** and **7f** can affect the cell viability of HT-1376 cell line, while they had no significant effect on the control ARPE-19 cells viability. Furthermore, none of the formulations tested are toxic in the absence of light to both HT-1376 and ARPE-19 cell lines, suggesting that the phototoxic effect is due to the reactive oxygen species production under irradiation. These promising results encourage further in vivo testing of TZ-POR derivatives as prototypes of future PDT agents.

## Abbreviations

Ce6Chlorin e6DPiBF1,3-diphenylisobenzofuranIBX2-iodoxybenzoic acidPDTPhotodynamic TherapyPSPhotosensitizerPVPpolyvinylpyrrolidonePVP-TPPpolyvinylpyrrolidone-tetraphenylporphyrin micellesPVP-TZ-PORpolyvinylpyrrolidone-triazole-porphyrin formulationTFAtrifluoroacetic acidTLCthin layer chromatographyTZ-PORtriazole-porphyrin derivative.

## Figures and Tables

**Table 1:** Results obtained for cell survival of HT-1376 and ARPE-19 cells incubated in the dark during 4 h with 10 µM of PSs PVP-TZ-POR **7a**–**f** and PVP-TPP and irradiated with white light for 40 min with an irradiance of 20 mW.cm^–2^.^a^

Entry	Figure	MTT reduction (% of control) HT-1376 (±SD)	MTT reduction (% of control) ARPE-19 (±SD)
1	PVP-TZ-POR **7a**	28.74 ± 2.74	40.80 ± 3.89
2	PVP-TZ-POR **7b**	69.50 ± 3.09	74.97 ± 2.85
3	PVP-TZ-POR 7**c**	20.46 ± 4.03	65.48 ± 3.70
4	PVP-TZ-POR 7**d**	24.30 ± 1.87	58.50 ± 3.15
5	PVP-TZ-POR 7**e**	42.75 ± 3.57	98.44 ± 3.35
6	PVP-TZ-POR 7**f**	54.34 ± 3.99	94.02 ± 2.51
7	PVP-TPP	63.84 ± 2.00	46.91 ± 3.85

^a^ Data are the mean value ± S.D. of at least three independent experiments performed in triplicates.

## Supplementary Materials

Photophysical characterization and photostability studies of TZ-POR **7a**–**f** and PVP-TZ-POR **7a**–**f** and chemical compounds characterization data (^1^H and ^13^C NMR) of TZ-POR **6a**–**f** and **7a**–**f** are provided. The results of cellular uptake and cell viability after PDT treatment of PVP-TZ-POR in HT-1376 and ARPE-19 cells not shown in the article are also presented.
