# Supplementary material for: Synthesis, Characterization and Photodynamic Activity against Bladder Cancer Cells of Novel Triazole-Porphyrin Derivatives
Source: Molecules. 2020 Mar 31;25(7):1607. doi: 10.3390/molecules25071607 (PMC7180931; doi:10.3390/molecules25071607)
Supplement: Supplementary file 1 [file molecules-25-01607-s001.pdf]

molecules

MDPI

Supplementary materials

Synthesis, Characterization and Photodynamic Activity Against Bladder Cancer Cells of Novel Triazole-Porphyrin Derivatives

Ana T. P. C. Gomes [1,*], Rosa Fernandes [2,3,*], Carlos F. Ribeiro [2,3], João P. C. Tomé [4], Maria G. P. M. S. Neves [1], Fernando de C. da Silva [5], Vítor F. Ferreira [6] and José A. S. Cavaleiro [1,*]

[1] LAQV-REQUIMTE, Department of Chemistry, University of Aveiro, 3810-193 Aveiro, Portugal; gneves@ua.pt (M.G.P.M.S.N.)
[2] Coimbra Institute for Clinical and Biomedical Research (iCBR), Faculty of Medicine, University of Coimbra, 3000-548 Coimbra, Portugal; cribeiro@fmed.uc.pt (C.F.R.)
[3] Center for Innovative Biomedicine and Biotechnology (CIBB), University of Coimbra, 3004-504 Coimbra, Portugal
[4] CQE, Departamento de Engenharia Química, Instituto Superior Técnico, Universidade de Lisboa, 1049-001 Lisboa, Portugal; jtome@tecnico.ulisboa.pt (J.P.C.T.)
[5] Departamento de Química Orgânica, Instituto de Química, Universidade Federal Fluminense, Niterói, 24020-150, RJ, Brazil; gqofernando@yahoo.com.br (F.d.C.d.S.)
[6] Departamento de Tecnologia Farmacêutica, Faculdade de Farmácia, Universidade Federal Fluminense, Niterói, 24241-000 RJ, Brazil; vitorferreira@id.uff.br (V.F.F.)
* Correspondence: ana.peixoto@ua.pt (A.T.P.C.G.); rcfernandes@fmed.uc.pt (R.F.); jcavaleiro@ua.pt (J.A.S.C.); Tel.: (optional; include country code; if there are multiple corresponding authors, add author initials) +xx-xxxx-xxx-xxxx (F.L.)

Table of contents

1. Photophysical characterization of TZ-POR 7a-f and formulations PVP-TZ-POR 7a-f....... 2

2. Photostability studies of formulations PVP-TZ-POR 7a–f 3

3. ^{1}H and ^{13}C NMR and HRMS spectra of TZ-POR 6a–f and 7a–f 4

3. Cellular uptake of PVP-TZ-POR 7a,c,d,f and PVP-TPP... 13

4. Cell viability of HT-1376 and ARPE-19 cells after PDT treatment with PVP-TZ-POR 7a-d, PVP-TPP and PVP 14

5. Dark toxicity of PVP-TZ-POR 7a-d, PVP-TPP and PVP in HT-1376 and ARPE-19 cells . 15

1. Photophysical characterization of TZ-POR 7a-f and formulations PVP-TZ-POR 7a-f

Table S1. Photophysical data of TZ-POR **7a–f** and formulations PVP-TZ-POR **7a–f.**

TZ-POR	λ_{max} (nm) (log ε)[a]	λ_{em} (nm)[a]	Stokes shift (nm)[a]	ϕ_F[b]	PVP-TZ-POR	λ_{max} (nm) (log ε)[a]	λ_{em} (nm)[a]	Stokes shift (nm)[a]	ϕ_F[b]
	424 (5.31)					424 (5.22)			
	522 (4.23)					521 (4.16)			
**7a**	561 (3.99)	659, 728	5	0.14	**7a**	560 (3.90)	659, 726	6	0.10
	599 (3.84)					598 (3.72)			
	654 (3.59)					653 (3.45)			
	424 (5.11)					424 (5.31)			
	521 (4.03)					521 (4.27)			
**7b**	560 (3.77)	659, 727	5	0.08	**7b**	560 (4.00)	659, 726	6	0.07
	598 (3.58)					598 (3.82)			
	654 (3.29)					653 (3.54)			
	423 (5.99)					422 (5.65)			
	521 (3.96)					521 (4.59)			
**7c**	561 (3.72)	660, 727	6	0.09	**7c**	560 (4.37)	657, 726	5	0.11
	598 (3.53)					598 (4.21)			
	654 (3.26)					652 (4.04)			
	423 (5.64)					423 (5.12)			
	521 (4.54)					521 (4.04)			
**7d**	560 (4.26)	659, 726	5	0.13	**7d**	559 (3.78)	659, 727	6	0.09
	598 (4.09)					598 (3.61)			
	654 (3.82)					653 (3.37)			
	423 (5.21)					423 (5.12)			
	521 (4.12)					521 (4.09)			
**7e**	560 (3.84)	659, 727	6	0.09	**7e**	559 (3.82)	659, 729	6	0.04
	598 (3.67)					598 (3.65)			
	653 (3.36)					653 (3.41)			
	423 (5.35)					424 (5.36)			
**7f**	521 (4.31)	659, 727	5	0.11	**7f**	521 (4.08)	660, 726	8	0.08
	560 (4.03)					560 (3.80)			

598 (3.86)	598 (3.61)
654 (3.58)	652 (3.28)

[a] in DMF/H2O (9:1)

[b] **TPP** in DMF as reference (ϕ_F = 0.12)

2. Photostability Studies of Formulations PVP-TZ-POR 7a–f

Table S2. Photostability of 10 μM of PVP-TZ-POR **7a–f**, after irradiation with white light at a fluence rate of 20 mW.cm^{-2} for different periods of time (0-60 min).*

PS	λ (nm)	Irradiation Time (min)						
		0	10	20	30	40	50	60
PVP-TZ-POR 7a	420	100	96	94	93	92	90	89
PVP-TZ-POR 7b	420	100	97	95	93	92	90	89
PVP-TZ-POR 7c	420	100	96	94	92	91	89	88
PVP-TZ-POR 7d	420	100	96	94	93	91	90	91
PVP-TZ-POR 7e	420	100	98	96	95	94	94	94
PVP-TZ-POR 7f	420	100	95	94	93	91	91	91
PVP-TPP	420	100	98	97	97	96	95	95

*The results are presented in percentage calculated by the ratio of residual absorbance at 420 nm at different periods of time and absorbance before irradiation.

3. ^{1}H and ^{13}C NMR and HRMS spectra of TZ-POR 6a–f and 7a–f

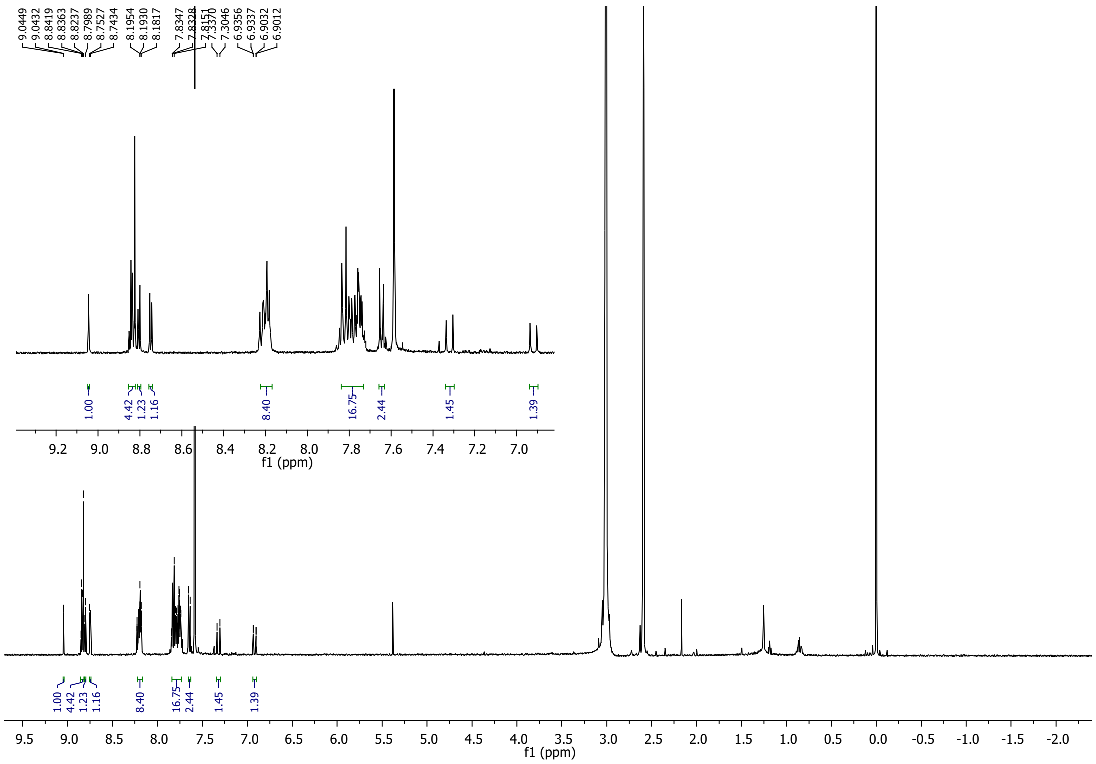

Figure S1. ^{1}H NMR of TZ-POR **6a** in $CDCl_3$.

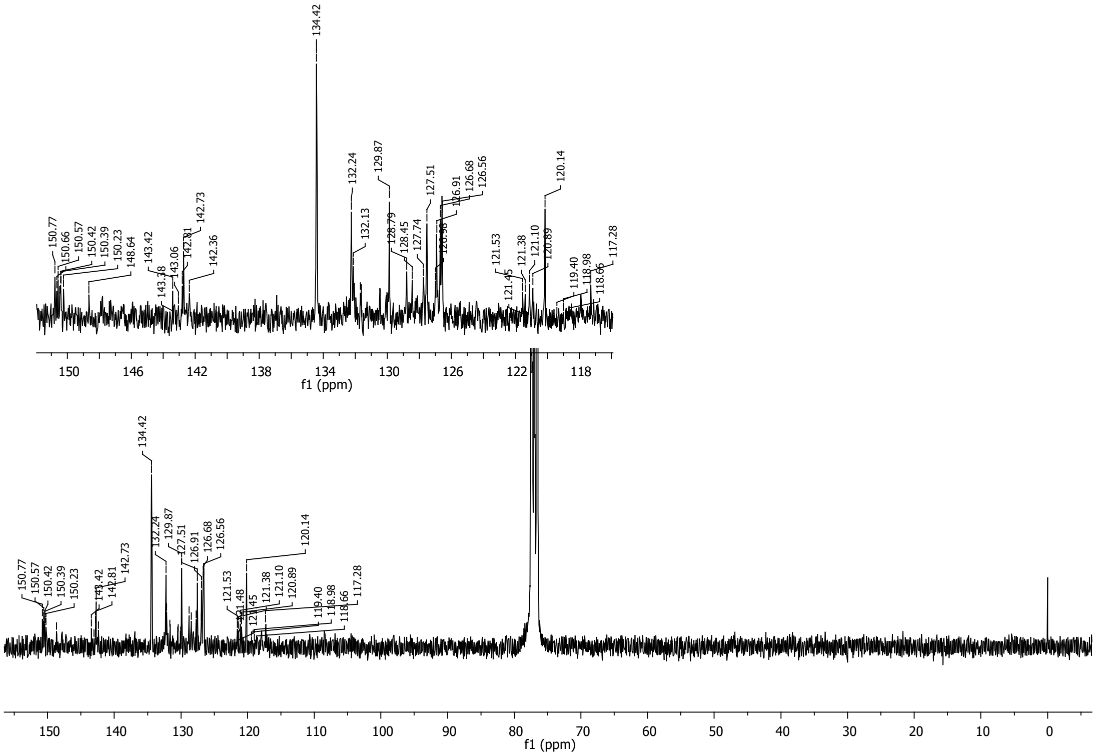

Figure S2. ^{13}C NMR of TZ-POR **6a** in $CDCl_3$.

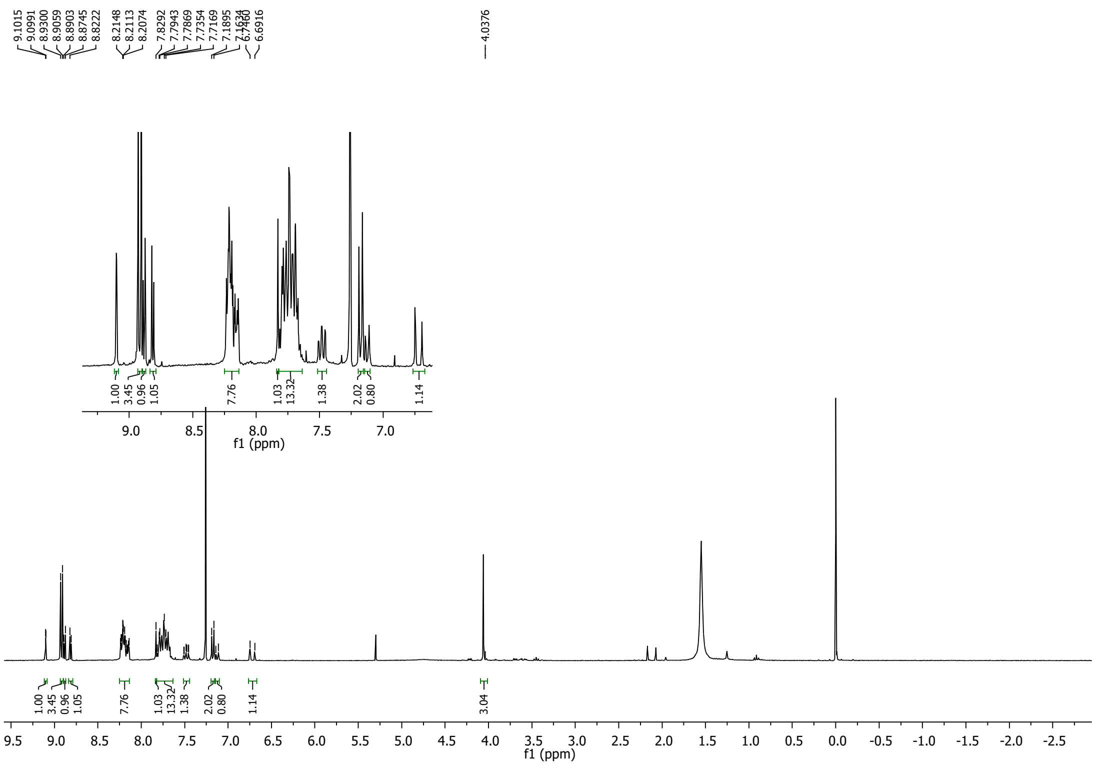

Figure S3. ^{1}H NMR of TZ-POR **6b** in $CDCl_3$.

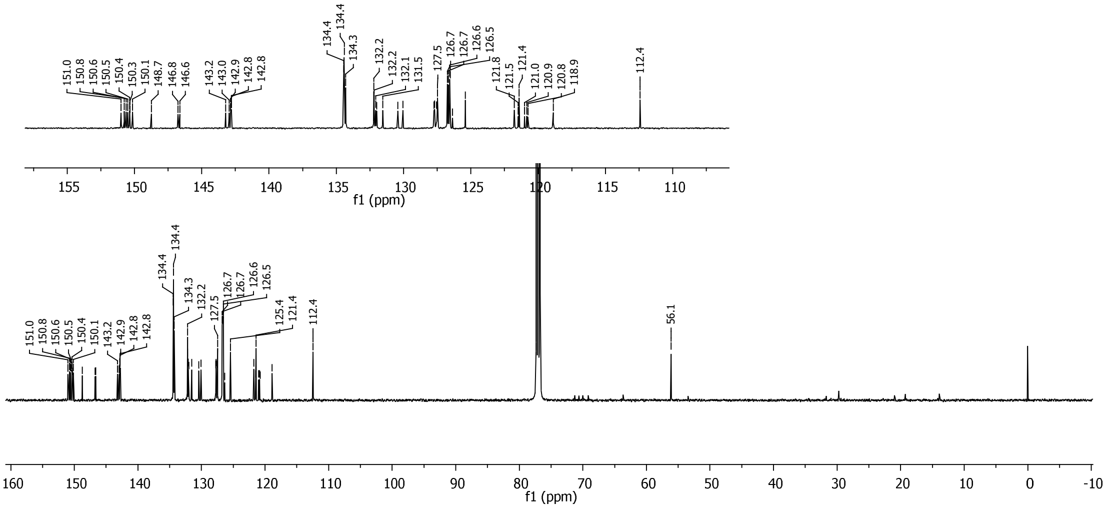

Figure S4. ^{13}C NMR of TZ-POR **6b** in $CDCl_3$.

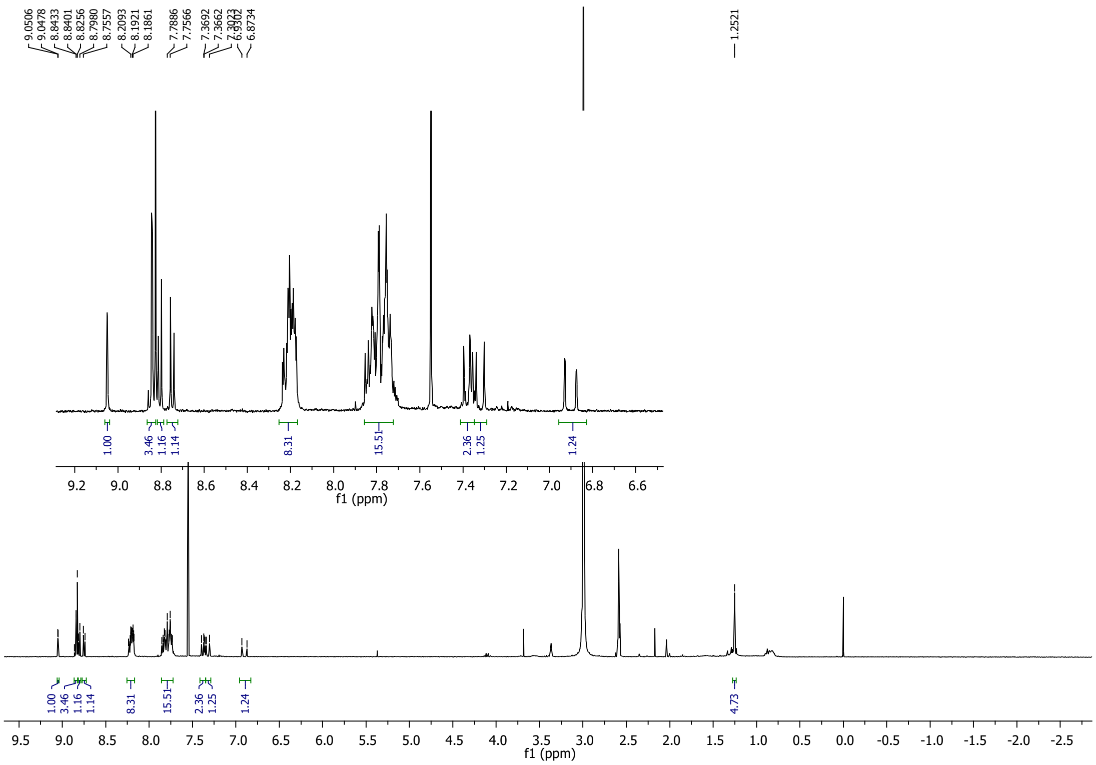

Figure S5. ^{1}H NMR of TZ-POR **6c** in $CDCl_3$.

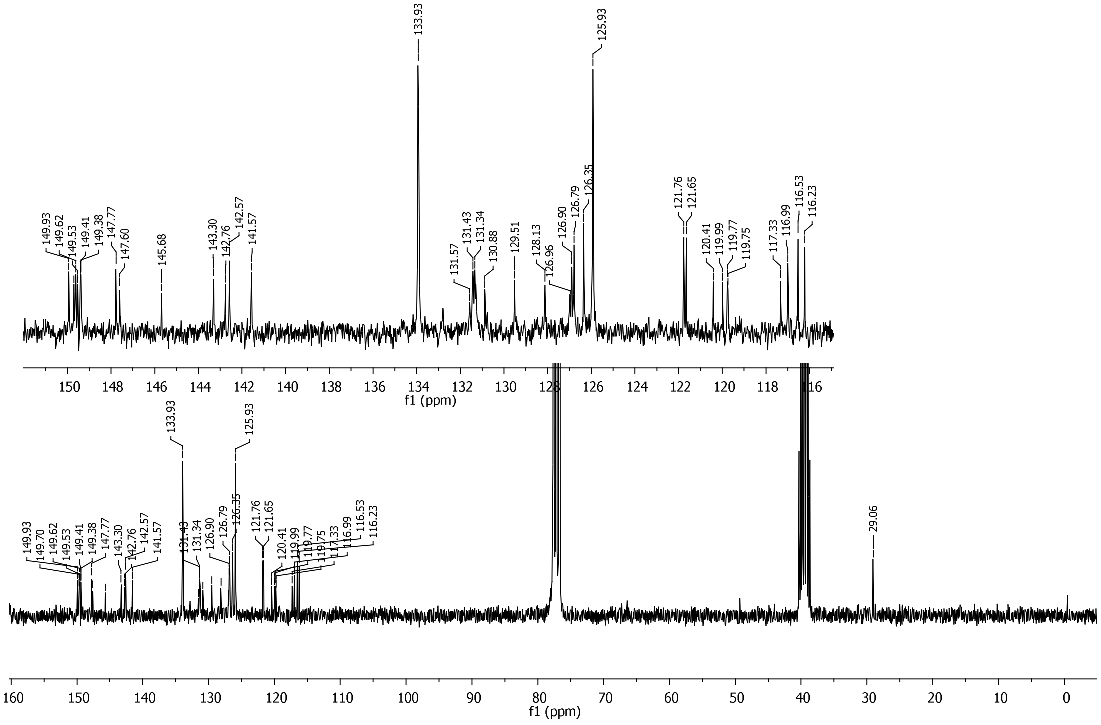

Figure S6. ^{13}**C NMR of** TZ-POR **6c** in $CDCl_3$ and DMSO-d_6.

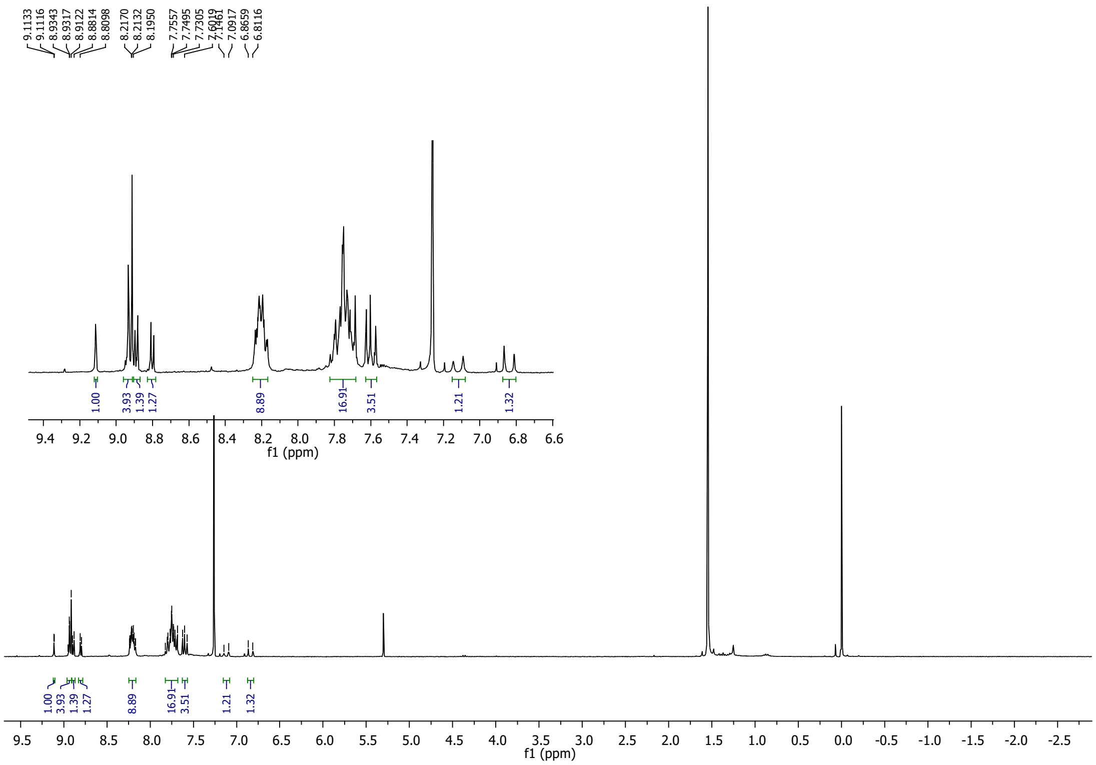

Figure S7. ^{1}H NMR of TZ-POR 6d in $CDCl_3$.

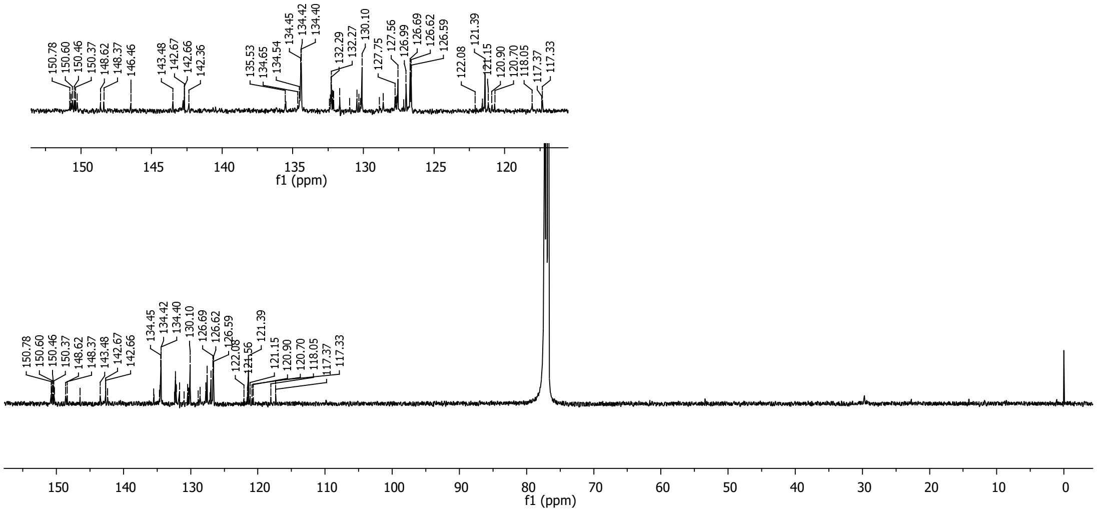

Figure S8. ^{13}C NMR of TZ-POR **6d** in $CDCl_3$.

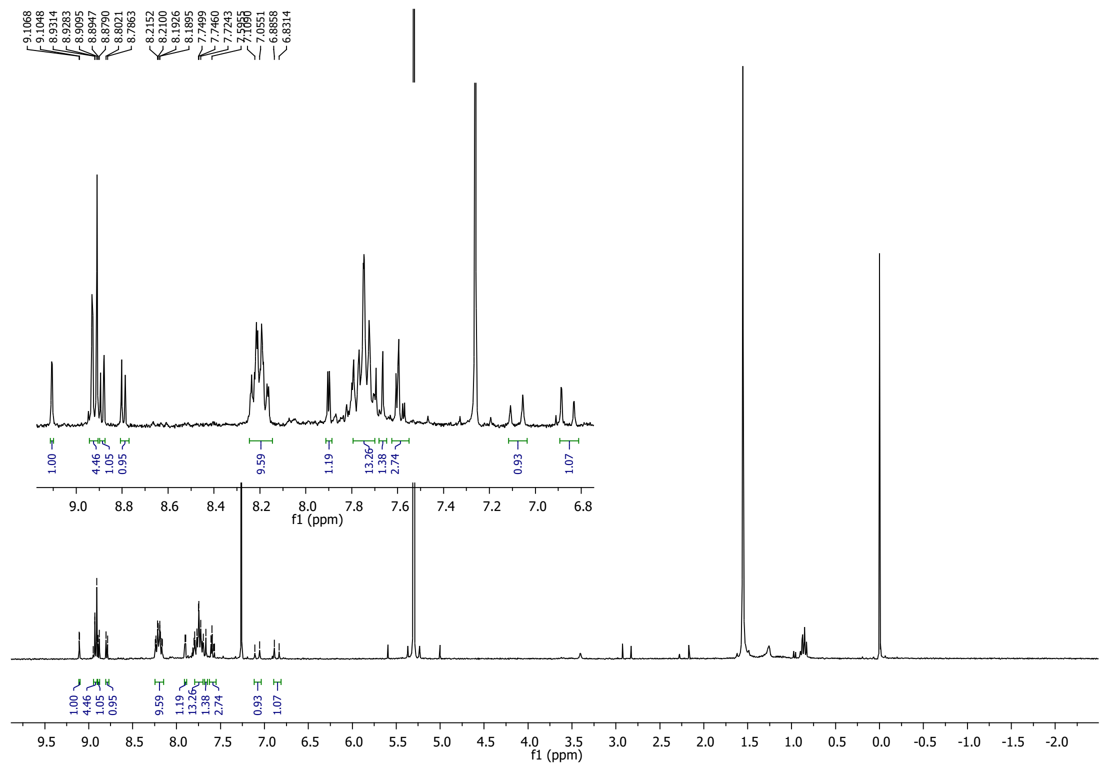

Figure S9. ^{1}H NMR of TZ-POR **6e** in $CDCl_3$.

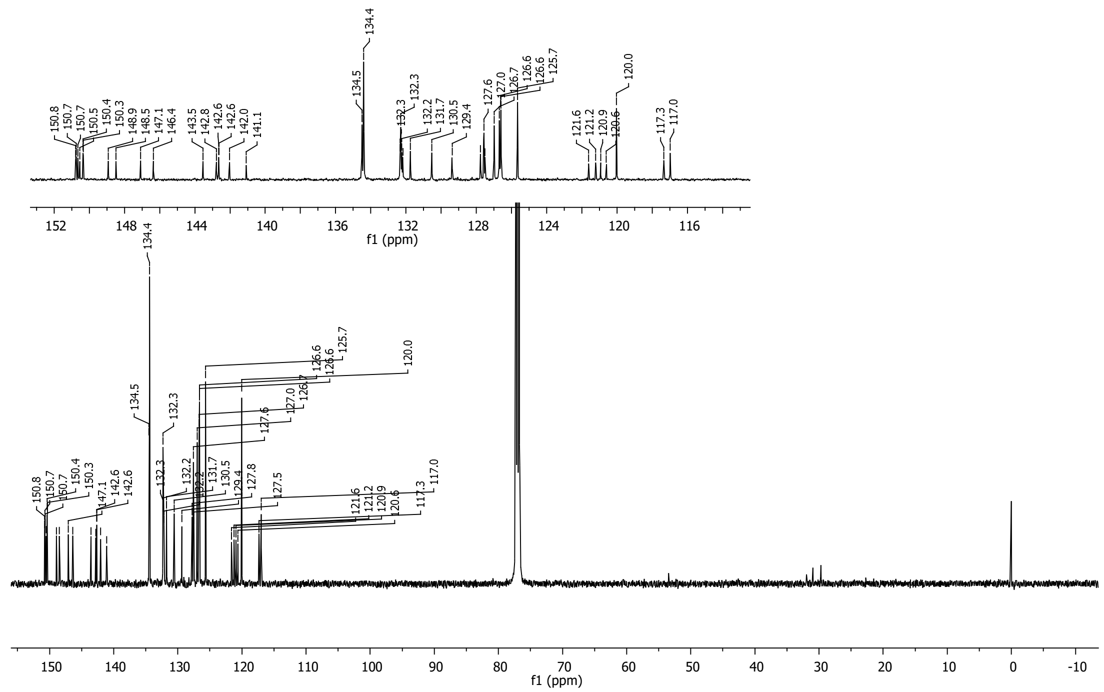

Figure S10. ^{13}C NMR of TZ-POR **6e** in $CDCl_3$.

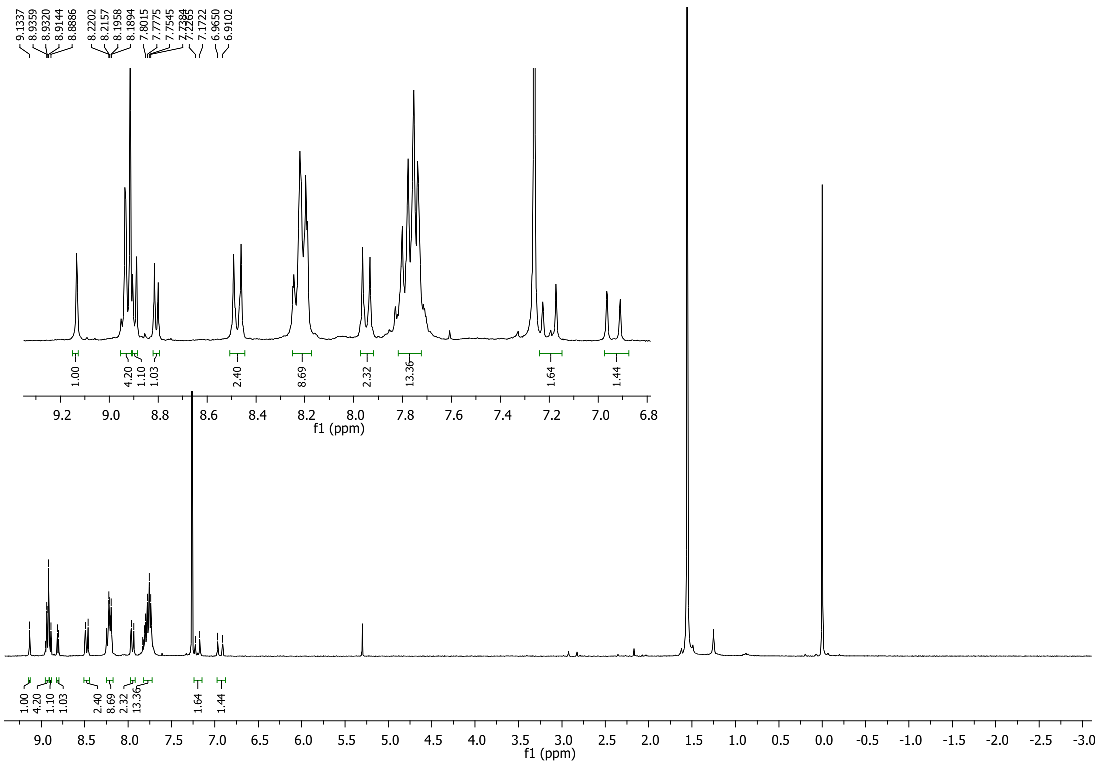

Figure S11. ^{1}H NMR of TZ-POR **6f** in $CDCl_3$.

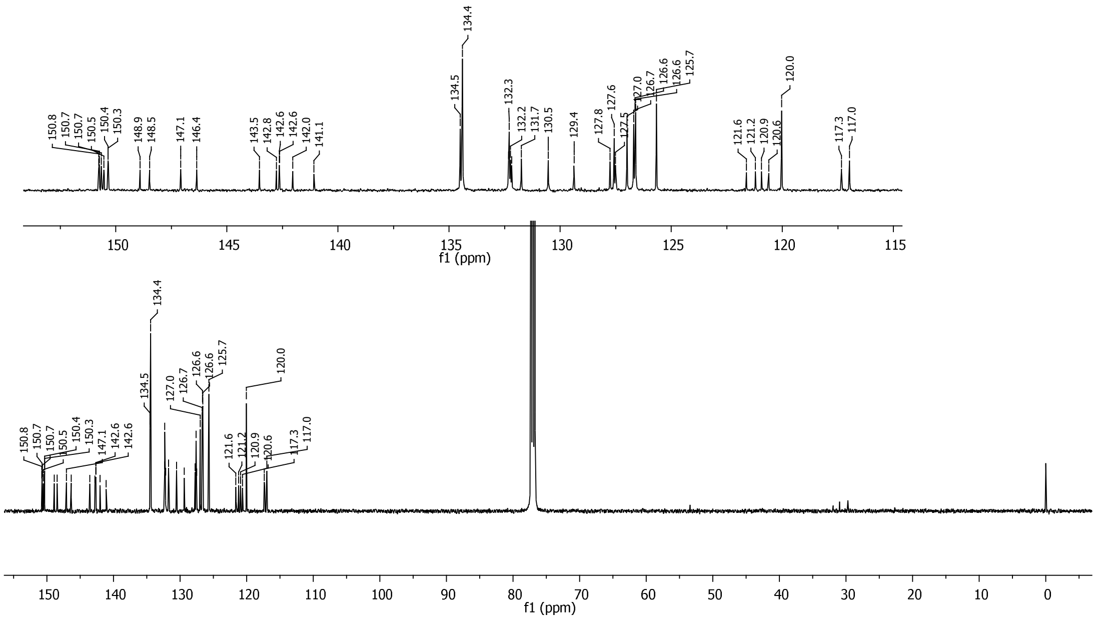

Figure S12. ^{13}C NMR of TZ-POR **6f** in $CDCl_3$.

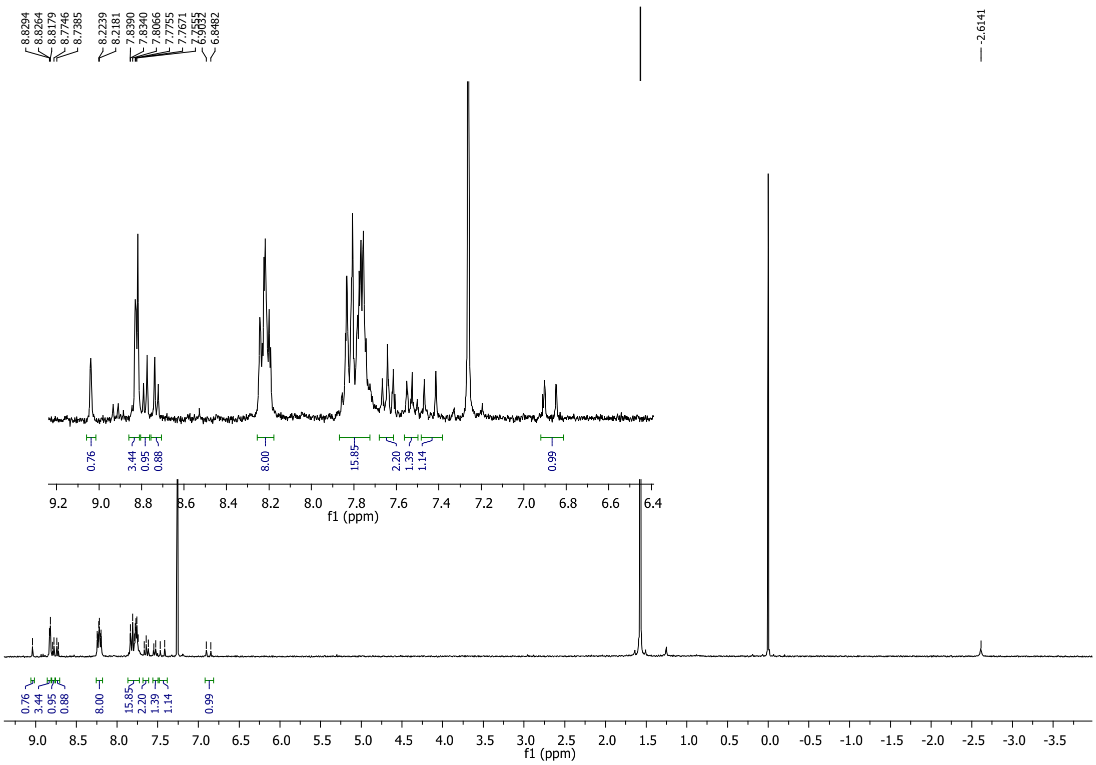

Figure S13. ^{1}H NMR of TZ-POR **7a** in $CDCl_3$.

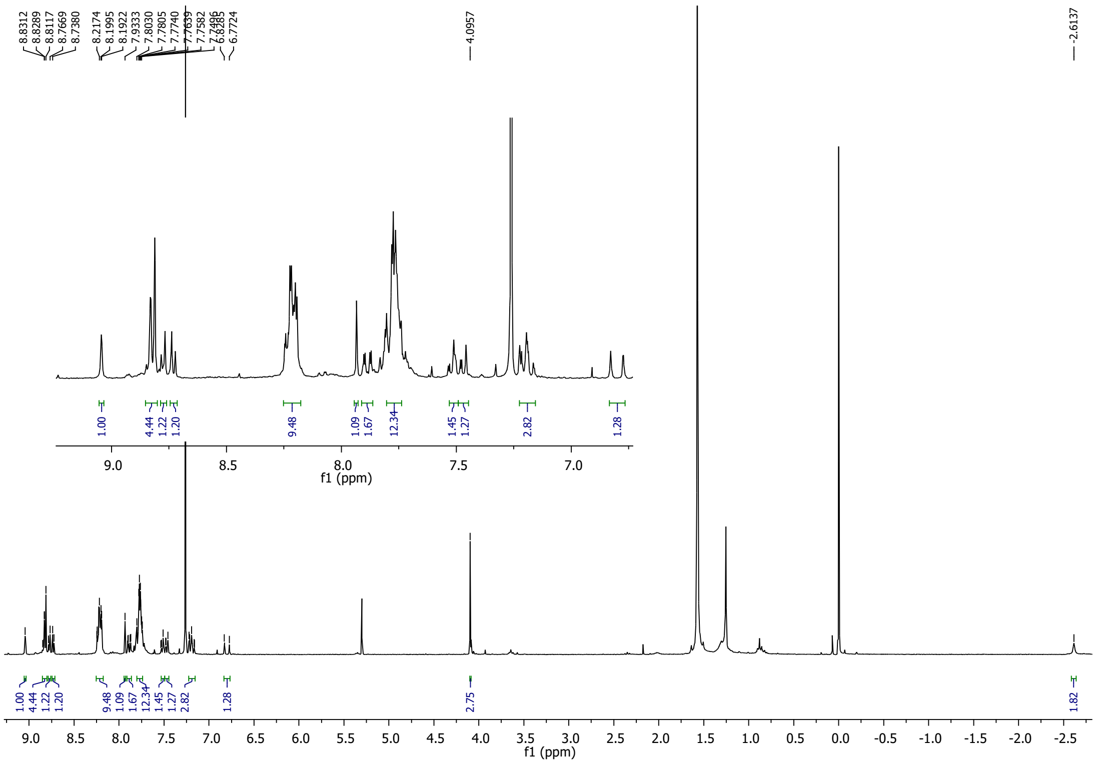

Figure S14. ^{1}H NMR of TZ-POR **7b** in $CDCl_3$.

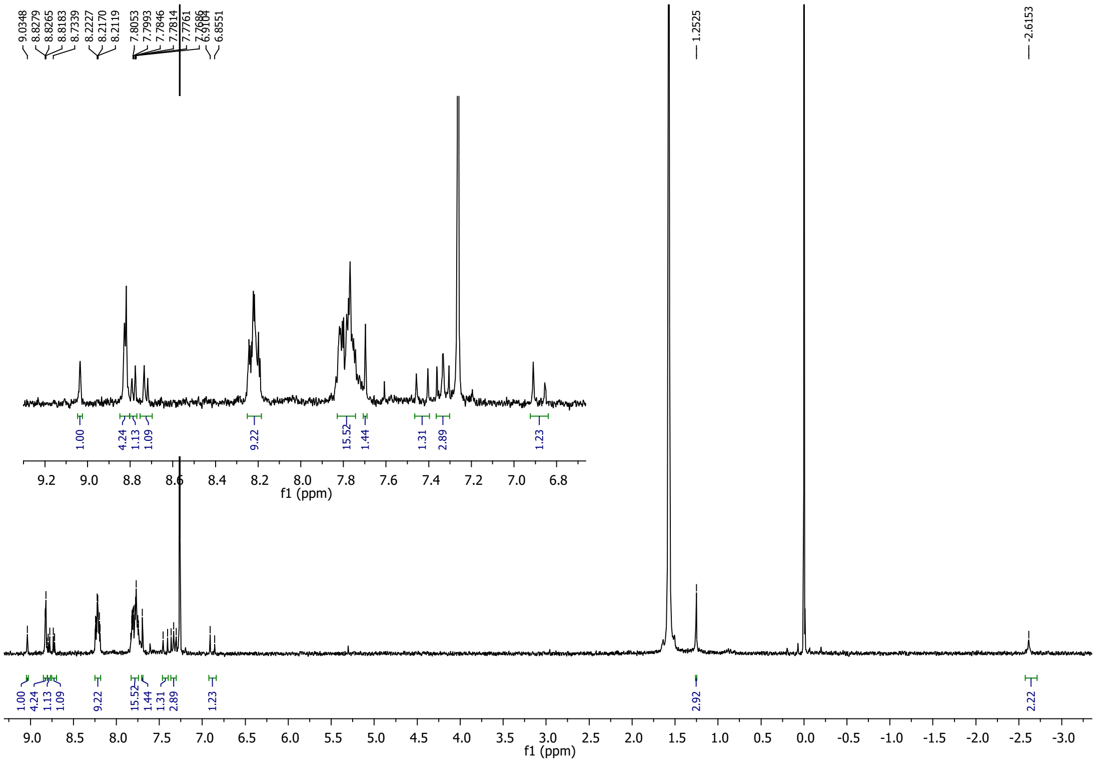

Figure S15. ^{1}H NMR of TZ-POR **7c** in $CDCl_3$.

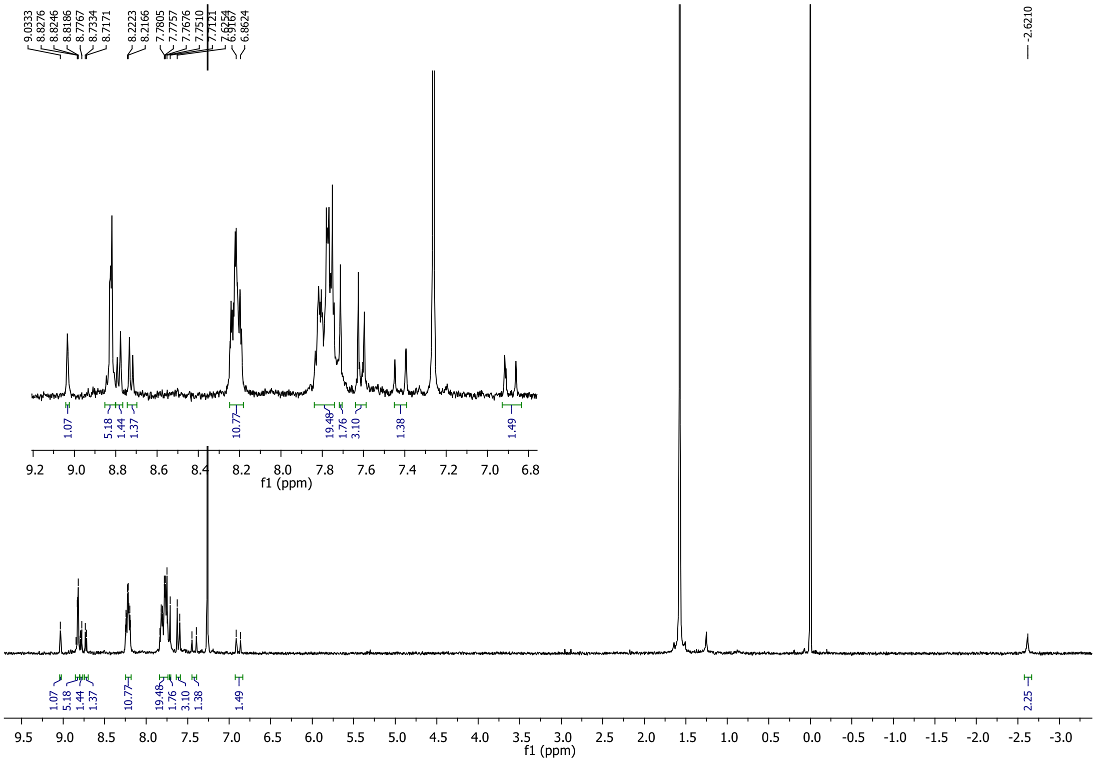

Figure S16. ^{1}H NMR of TZ-POR **7d** in $CDCl_3$.

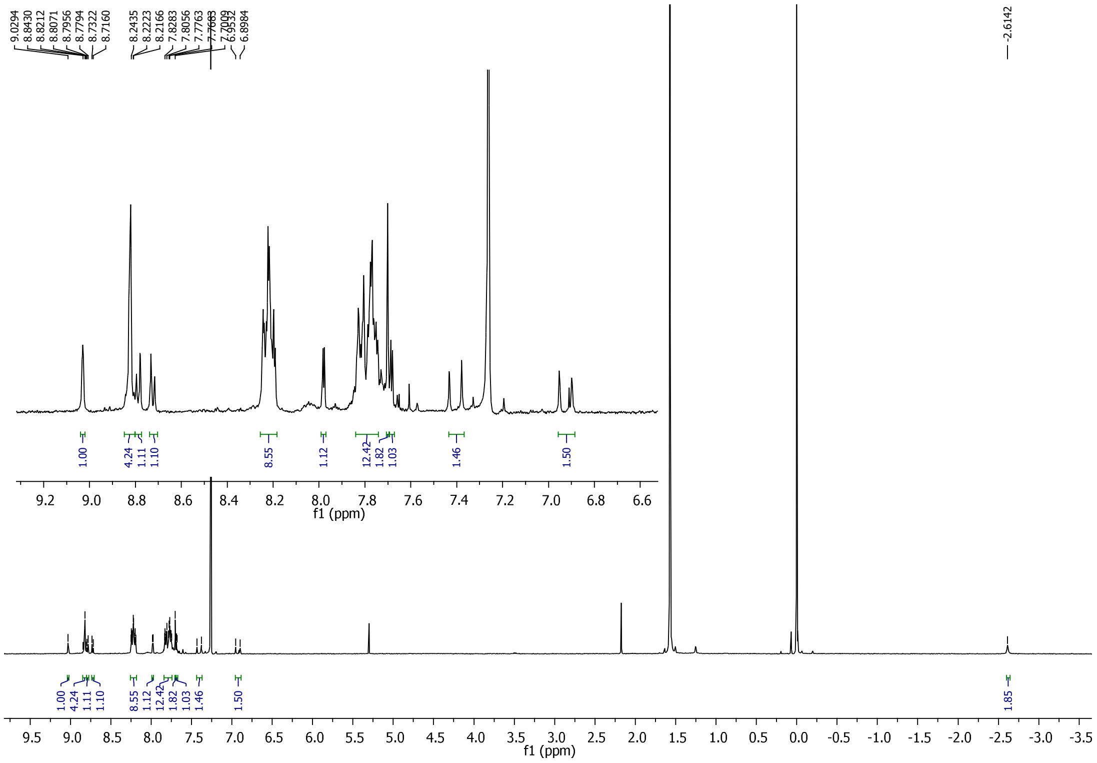

Figure S17. ¹H NMR of TZ-POR **7e** in $CDCl_3$.

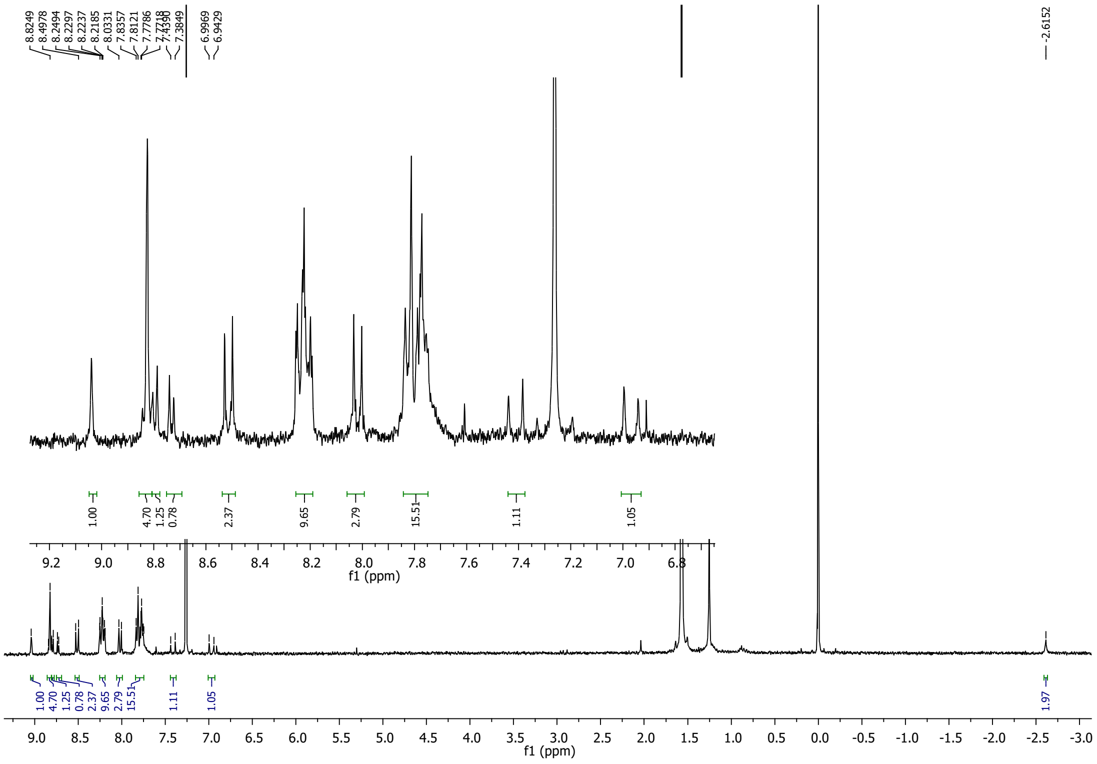

Figure S18. ¹H NMR of TZ-POR **7f** in $CDCl_3$.

3. Cellular Uptake of PVP-TZ-POR 7a,c,d,f and PVP-TPP

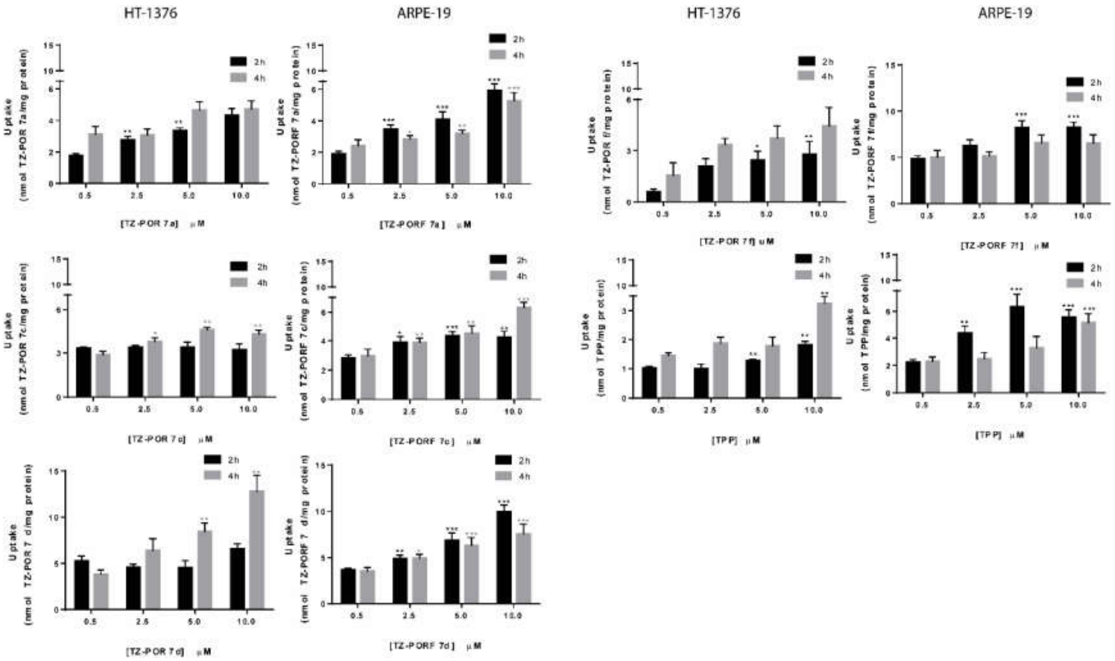

Figure S19. Intracellular uptake of PVP-TZ-POR **7a,c,d,f** and PVP-TPP by HT-1376 and ARPE-19 cells. Data are the mean ± S.D. of at least three independent experiments performed in triplicates. *($p < 0.05$), **($p < 0.01$), ***($p < 0.001$), significantly different from uptake of PSs.

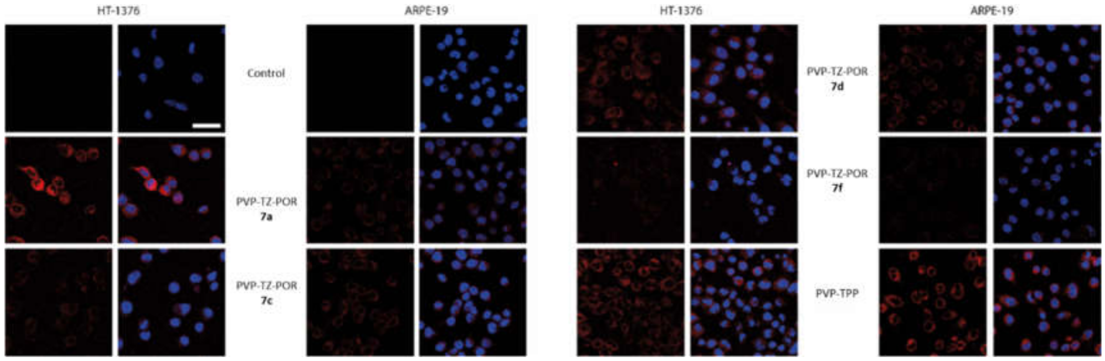

Figure S20. Representative fluorescence images of HT-1376 and ARPE-19 cell lines incubated with 10 μM of PSs PVP-TZ-POR **7a,c,d,f** and PVP-TPP (red) for 4 h in darkness and cell nucleus stained with DAPI (blue). *Scale bars* 20 μm.

4. Cell Viability of HT-1376 and ARPE-19 Cells After PDT Treatment with PVP-TZ-POR 7a-d, PVP-TPP and PVP

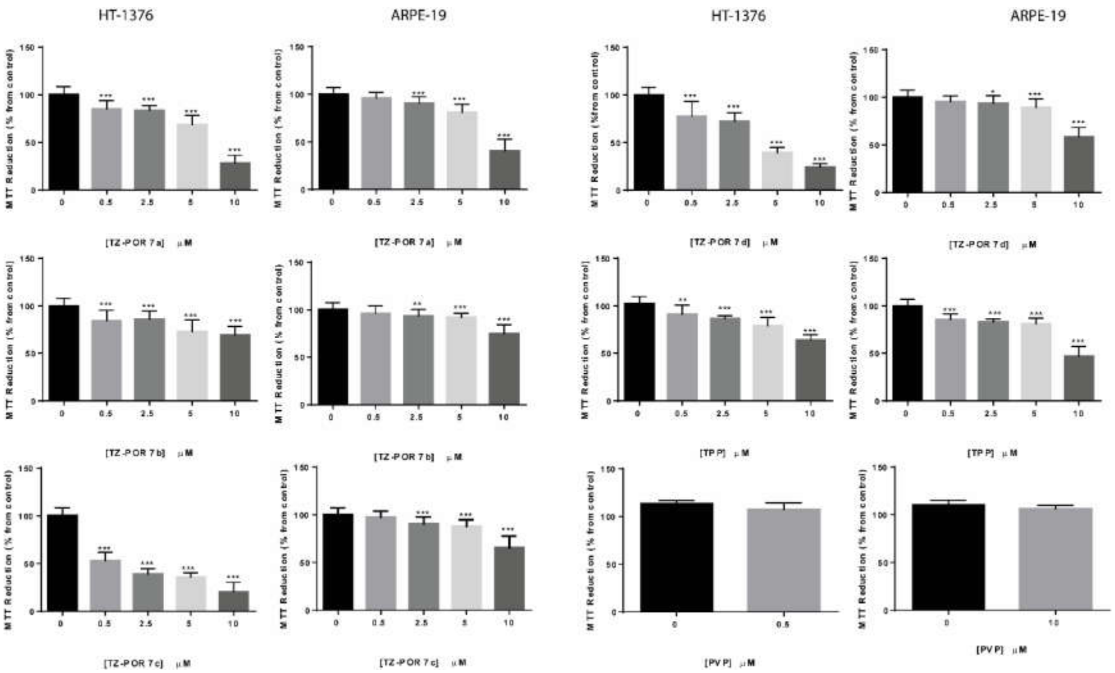

Figure S21. Cell survival of HT-1376 and ARPE-19 cells, incubated in the dark during 4 h with PSs PVP-TZ-POR **7a-d,** PVP-TPP and PVP at different concentrations and irradiated with white light for 40 min with an irradiance of 20 mW.cm^{-2}. Data are the mean value ± S.D. of at least three independent experiments performed in triplicates. *($p < 0.05$), **($p < 0.01$), ***($p < 0.001$), significantly different from MTT reduction (%).

5. Dark toxicity of PVP-TZ-POR 7a-d, PVP-TPP and PVP in HT-1376 and ARPE-19 cells

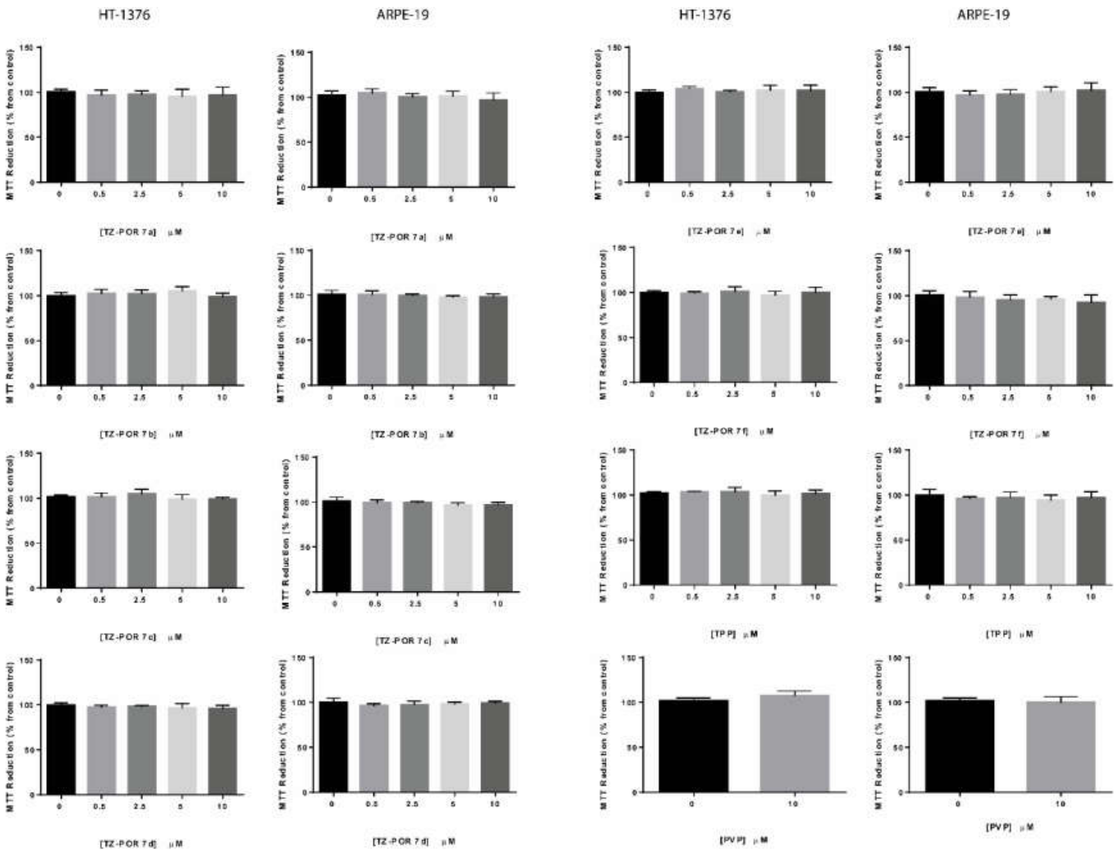

Figure S22. Cell survival of HT-1376 and ARPE-19 cells, incubated in the dark during 4 h with PSs PVP-TZ-POR **7a-f**, PVP-TPP and PVP at different concentrations. Data are the mean value ± S.D. of at least three independent experiments performed in triplicates.
